# Descriptor-augmented machine learning for enzyme-chemical interaction predictions

**DOI:** 10.1016/j.synbio.2024.02.006

**Published:** 2024-02-28

**Authors:** Yilei Han, Haoye Zhang, Zheni Zeng, Zhiyuan Liu, Diannan Lu, Zheng Liu

**Affiliations:** a Department of Chemical Engineering, Tsinghua University, Beijing, 100084, China; b Department of Computer Science and Technology, Tsinghua University, Beijing, 100084, China

**Keywords:** Enzyme-substrate interaction, Enzyme design, Machine learning, Descriptor

## Abstract

Descriptors play a pivotal role in enzyme design for the greener synthesis of biochemicals, as they could characterize enzymes and chemicals from the physicochemical and evolutionary perspective. This study examined the effects of various descriptors on the performance of Random Forest model used for enzyme-chemical relationships prediction. We curated activity data of seven specific enzyme families from the literature and developed the pipeline for evaluation the machine learning model performance using 10-fold cross-validation. The influence of protein and chemical descriptors was assessed in three scenarios, which were predicting the activity of unknown relations between known enzymes and known chemicals (new relationship evaluation), predicting the activity of novel enzymes on known chemicals (new enzyme evaluation), and predicting the activity of new chemicals on known enzymes (new chemical evaluation). The results showed that protein descriptors significantly enhanced the classification performance of model on new enzyme evaluation in three out of the seven datasets with the greatest number of enzymes, whereas chemical descriptors appear no effect. A variety of sequence-based and structure-based protein descriptors were constructed, among which the esm-2 descriptor achieved the best results. Using enzyme families as labels showed that descriptors could cluster proteins well, which could explain the contributions of descriptors to the machine learning model. As a counterpart, in the new chemical evaluation, chemical descriptors made significant improvement in four out of the seven datasets, while protein descriptors appear no effect. We attempted to evaluate the generalization ability of the model by correlating the statistics of the datasets with the performance of the models. The results showed that datasets with higher sequence similarity were more likely to get better results in the new enzyme evaluation and datasets with more enzymes were more likely beneficial from the protein descriptor strategy. This work provides guidance for the development of machine learning models for specific enzyme families.

## 1 Introduction

Green chemistry constantly pursues enzymes with high activity and selectivity [1]. The advance of high throughput enzyme activity assay techniques [2] combined with proficient enzyme engineering, design, and selection algorithms [3] has paved the way for efficient creation of novel enzymes. However, the complex enzyme-chemical interactions [4] and various molecular mechanisms [5] in enzyme catalysis pose great challenges to theory-based construction process for new enzymes, particularly those intended to work under non-natural settings [6].

Enzyme substrate promiscuity characterizes an enzyme's reactivity to non-natural substrates. It implies that an enzyme can catalyze a wide range of substances, not simply its native neighbors [7]. This concept has gained popularity in recent years [8]. In metabolic engineering, substrate promiscuity depicts many-to-many reaction profiles that replace simplistic linear reaction cascade models with more realistic reaction network models, allowing engineers to design and regulate *in vivo* synthetic pathways more precisely [9,10]. In in vitro applications, catalysts based on enzymes with high substrate promiscuity may be capable of handling more diverse compounds [11]. As a result, identifying promiscuous activities of enzymes has become a trend in the development of novel biocatalysts [12].

Several functionally related enzyme families have had their substrate profiles studied [[13], [14], [15], [16], [17], [18], [19], [20]]. Meanwhile, prediction models for enzymes and chemicals are being built to explain the pattern in the activity matrix. Some earlier methods tended to make classification using only a few key reactivity indicators. Bastard et al. [16] explored the unprobed sequence space to fully annotate the function of the newly discovered β-ketoacid lyase family. They proposed the active sites modeling and clustering concept which employed the 20 amino acids near the enzyme's active site as a simplified sequence to represent the full enzyme. This shortened sequence effectively distinguished enzymes with varying substrate preference. Fisher et al. [18] targeted halogenation enzymes for C-H bond functionalization and screened flavin-dependent halogenases for chemo-selectivity and site-selectivity. Enzymes were sampled using the sequence similarity network. To explain the observed activity profile, the halogenation free energy changes at different locations of the compounds were estimated using quantum chemistry methods. Guallar et al. [19] guided a large-scale esterase screening to find enzymes with substrate promiscuity comparable to CALB. In this study, the active site effective volume based on enzyme substrate pockets was suggested as a predictor of esterase promiscuity. It could identify highly promiscuity esterases from low promiscuity esterases with an accuracy of 94%. Despite their high stated accuracy for qualitative tasks, these approaches take into account fewer aspects, which may make them inappropriate for more precise applications, such as precisely predicting an enzyme's substrate profile [21].

Machine learning is effective at inferring patterns from complex data, making it popular in the field of protein engineering [[22], [23], [24]]. It has also been used to predict enzyme-chemical activity relationships [[25], [26], [27]]. Yang et al. [25] were the first to build a machine learning model based on enzyme-chemical activity screening results. They assayed the activity of the glycosyltransferase GT1 family from the Arabidopsis thaliana genome to glycosyl donors and acceptors and created a substrate prediction model GT-Predict. GT-Predict was a two-stage model. It built a classifier for each known enzyme to evaluate the activity of chemicals. For a new enzyme, it used sequence alignment to obtain the top-k most similar enzymes and used the weighted average score of their chemical evaluation scores for suggestion. The most useful chemical properties in this strategy include physicochemical properties such as LogP, surface area, volume, chemical category and so on. Predictions of substrate profiles for enzymes from the genomes of two other organisms were used to validate the model. Ole-A thioesterases [26] and nitrilases [27] are two recent case studies. Robinson et al. [26] trained machine learning models with descriptors of both enzymes and substrates as input. For enzymes, the descriptors were the physicochemical properties of amino acid residues close to the active site, and for chemicals, the computed physicochemical factors. The use of well-designed descriptors improved model accuracy in predicting enzyme substrate relationship. Mou et al. [27] incorporated more descriptors into their machine learning model. They included the physicochemical parameters of nitrilase active site, nitrile substrate physicochemical properties, nitrile substrate reactivity computed using quantum chemistry and the energy terms from the enzyme-substrate docking conformations. The results indicated that structural models and docking conformations could provide more precise descriptions.

These studies collectively demonstrated the efficacy of prediction models for enzyme-chemical relationships when the enzyme and chemical properties are fairly considered. However, due to the specificity of the models, it could be difficult to draw general conclusions about the extent to which current strategies are effective and what factors are crucial for prediction [28]. Machine learning provides the opportunity to examine this problem within the context of a consistent framework, universal approaches, and rigorous evaluation [29,30].

In this study, we investigated the effect of protein and chemical descriptors on the prediction of enzyme-substrate relationships in three scenarios relevant to practical applications using the activity datasets of specific enzyme families. Our study was carried out separately for each enzyme family, but the evaluation technique and descriptor construction are consistent. For enzymes, we started with the sequences and used established tools to prepare descriptors to explore the impact of introducing different enzymes traits such as physicochemical properties, sequence correlation, and structural stability, among others. For chemicals, we use a collection of descriptors to describe their structural, topological, and physicochemical properties. We undertake detailed ablation experiments at both the descriptor and model levels to demonstrate the true influence of the descriptors. Finally, we hope to test the generality of the existing approach by correlating the statistical characteristics of the datasets with model performance.

## 2 Methods

### 2.1 Data curation

The datasets were collected from the original literature [16,[18], [19], [20],[25], [26], [27]] and the metadata of the datasets were recorded in Supplementary Table S1. For activity data, the original continuous values were taken from the literature. In some pipelines, the data are transformed by log function or binarization function. For chemicals, the SMILES linear annotation or 2D structures were taken from the main text, the supplementary files, or the online data source of the literature. And then 3D structures were obtained from the Pubchem online server or converted from SMILES by *RDKit*. For enzymes, the sequences or the UniProt or NCBI accessions were taken from the original files of the literature.

**Fig. 1:**
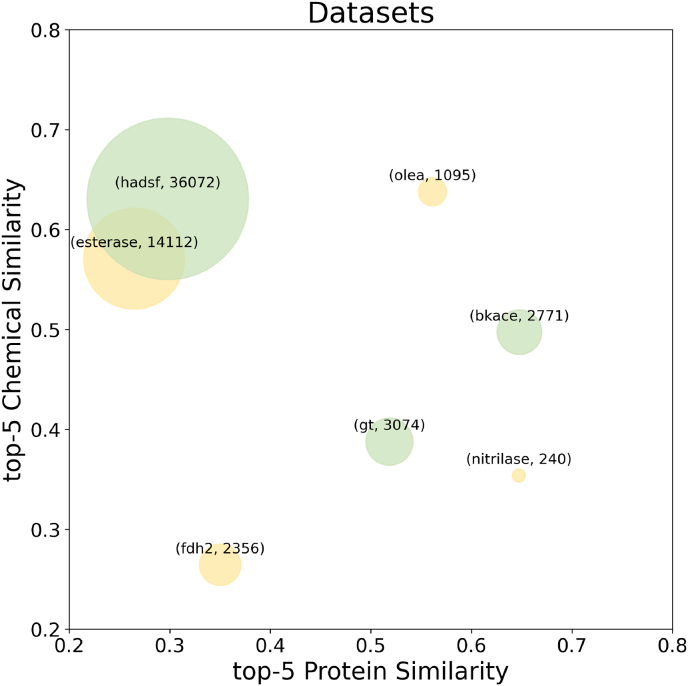
**Bubble plot of the datasets.** The x-axis represents the mean of the top-5 protein similarity of the datasets, while the y-axis represents the mean of the top-5 chemical similarity. Each bubble's size corresponds to the number of experimental data points in the dataset (the product of the number of enzymes and the number of chemicals). The color of the bubbles represents the source of the substrate library: green represents endogenous metabolites of cells and yellow represents synthetic chemicals. The following number of enzymes and chemicals are represented in the seven datasets: esterase (147, 96), hadsf (216, 167), bkace (163, 17), nitrilase (12, 20), olea (73, 15), and fdh2 (38, 62), gt (53, 58).

As illustrated in Fig. 1, current datasets cover diverse reaction types and data sizes, as well as varying substrate similarities and protein similarities. The datasets were classified as industrial ones or physiological ones depending on the substrate library screened. The former primarily characterized on non-natural substrates and the latter on natural metabolites. The datasets and curation scripts could be downloaded at https://github.com/ZOOEEER/ECI-data.

### 2.2 Statistics of the datasets

To characterize the datasets, some indicators are proposed to describe the distribution of the data set. Indicators could be considered from three aspects, related to activity data, enzymes, or chemicals respectively.

(1) Related to activity data:
  • ***Activity ratio***: For 0-1 activity data, it represents the proportion of active combinations; for continuous activity values, it represents the average activity of the enzyme family against the chemical library.
(2) Related to enzymes:
  • ***Number of enzymes***: The number of enzymes in the dataset used to evaluate the model for the specific task. For new relationship evaluation or new enzyme evaluation, it is equal to the number of enzymes in the data set. For new chemical evaluation, since enzymes with too high or too low activity are removed, it is smaller than the original number.
  • ***AOIE***: Activity Order Index at Enzyme axis. This number is calculated from activity data that reflects the degree to which the order of activity evaluated for enzymes on a chemical is consistent with the order of activity for the enzymes on the whole chemical library. For randomly generated activity data, the value is close to 0.5. The closer the value is to 1, the stronger the above-mentioned consistency, which means the activity order of the enzyme family for different chemicals is consistent. In other words, this enzyme family is less chemically specific.
  •***Top-5 protein similarity***: This number reflects the degree of similarity of the enzymes of the data set. First, similarity scores are calculated for all enzymes in the data set; then the top-5 scores of each enzyme are averaged, and then averaged across all enzymes. Enzyme similarity was determined using the Smith-Waterman algorithm for sequence similarity [29].
(3) Related to chemicals:
  • ***Number of chemicals***: The number of chemicals in the dataset used to evaluate the model for the specific task. For new relationship evaluation or new chemical evaluation, it is equal to the number of chemicals in the data set. For new enzyme evaluation, it is smaller than the original number.
  • ***AOIC***: Activity Order Index at Chemical axis. From the same flavor as the value defined at the enzyme axis, this value is 0.5 for completely random data and 1 for a library of chemicals that are not enzymatic-specific at all.
  • ***Top-5 chemical similarity***: This number reflects the degree of similarity of the chemicals of the data set. Its definition is the same as the similarity of enzymes. The one-to-one chemical similarity used here is the Tanimoto similarity of chiral 2048-bit Morgan fingerprints implemented in the *RDKit* package in python.

The statistics of the dataset are used to examine the generalization of the proposed machine learning pipeline by analyzing its correlations to the evaluation metrics of the models on different tasks. The implementation of these statistics is given in Supplementary Method S1. The analysis was done using *stats.pearsonr* function of the *scipy* package in Python.

### 2.3 Machine learning pipeline

#### 2.3.1 Dataset splitting

**Fig. 2:**
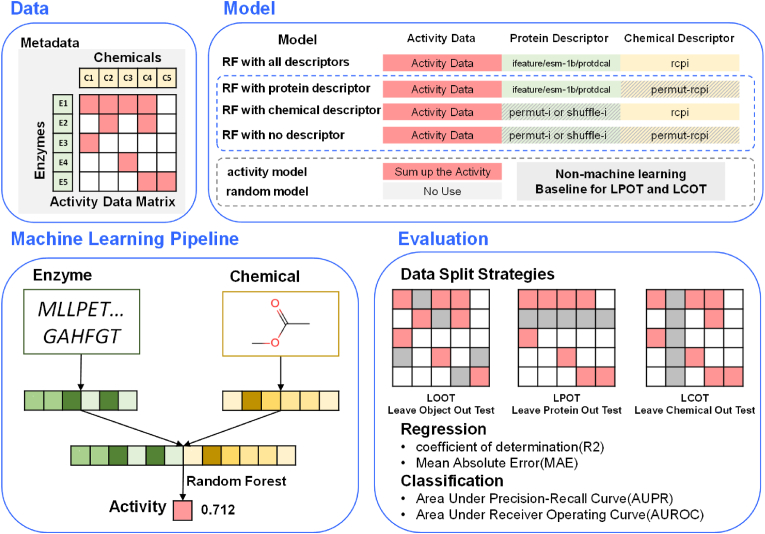
**The overall framework.** The enzyme and chemical relationship prediction process includes four key elements: (1) Data. One dataset includes enzymes, chemicals, activity data matrix and the metadata. (2) Machine Learning Pipeline. The pipeline takes the enzyme sequence and chemical structure as the original input. It uses the established software, such as ifeature, to embed the enzyme sequence as a numerical vector and use Rcpi to calculate the descriptor vector for the chemical. The two vectors are concatenated together to form the input of the machine learning model. The machine learning model here uses the Random Forest model. It outputs the predicted scores for subsequent evaluation. (3) Model. Here are seven types of models, which differ in their use of activity data, enzyme descriptors and chemical descriptors. RF with all descriptors uses all information as a standard model. RF with protein/chemical descriptors randomizes the chemical or protein descriptors based on the standard model, indicating that this information is not used. RF with no descriptor indicates that chemical and protein descriptors are randomized, indicating that both protein and chemical information are not used. Two models are used as baseline models for LPOT and LCOT: Activity model uses activity data without machine learning pipeline. Random model does not use the activity data. (4) Evaluation. Three data splitting strategies were conducted for cross-validation: Leave Object Out Test (LOOT), Leave Protein Out Test (LPOT), and Leave Chemical Out Test (LCOT), corresponding to evaluation of new relationships, evaluation of new enzymes, and evaluation of new chemicals, respectively. Here reports coefficient determination (R2) and mean absolute error (MAE) as metrics for regression and Area Under Precision-Recall curve (AUPR) and Area Under Receiver Operating Curve (AUROC) for classification.

Ten-fold cross-validation (CV) is used to evaluate the machine learning pipeline (see Fig. 2). The process is as follows: divide all the activity data into 10 folds equally, take a fold each time as the validation set, and the remaining 9 folds as the training set. Use the training set to train the model and evaluate the model on the validation set. The average and variance of the metrics on the 10 folds is reported for one CV. The process was done using *KFold* function of the *scikit-learn* package.

The splitting strategy of the dataset reflects how the model would be used in real scenarios [27,31,32]. And it would greatly affect the credibility of the results because of issues such as data breaches. In enzyme design problems, there are four scenarios, namely accessing the activity of known/unknown enzymes on known/unknown chemicals. The totally novel combination of enzymes and chemicals is out of scope of our data-driven method. Here, three data-splitting strategies have been established to simulate three of the scenarios.

• ***LOOT***: Leave Object Out Test or new relationship evaluation. All activity data (i.e., the activity assay results of pairwise combinations of all the enzymes and compounds) were randomly split into 10 folds, and a fold (10% of all data) was taken in turn as the validation set, and the rest (90% of all data) was used as the training set.
• ***LPOT***: Leave Protein Out Test or new enzyme evaluation. Split the enzymes randomly into 10 folds, take out a fold (10% of the enzyme) in turn to predict the activities of all chemicals on these enzymes and use the activity data of the remaining enzymes on all chemicals for training. To avoid data imbalance, the chemicals were filtered, i.e., only chemicals with an activity number between 10% and 90% of the number of enzymes and larger than 1 were retained [29].
• ***LCOT***: Leave Chemical Out Test or new chemical evaluation. Like LPOT, 10% of the chemicals are taken out each time as the validation set, and the rest are used as the training set. Similarly, filter the enzymes and only retain those whose activity number is between 10% and 90% of the number of chemicals and larger than 1.

Dataset splitting is controlled by the random number seed. To make the results more reliable, five random number seed have been used to do five parallel experiments for each task on each dataset. The mean and variance of the metrics were calculated as the final evaluation of the model.

#### 2.3.2 Evaluation metrics

For new relationship evaluation, both regression and classification problems were evaluated. For new enzyme evaluation and new chemical evaluation, model did not perform well on regression problems, so only the classification problems were evaluated. To compare different models, predictions on validation sets have been accessed using the following metrics.

For regression problems.

•***R2***: The squared Pearson correlation coefficient (R2) between predicted and observed activities is unitless and ranges from 0 to 1.
•***MAE***: The mean-absolute error (MAE) measuring the difference between predicted and observed values.

For classification problems.

•***AUPR***: Precision-Recall is a useful measure of success of prediction when the classes are very imbalanced, which is common in the field of protein engineering [29]. Precision is a measure of result relevancy, while recall is a measure of how many truly relevant results are returned. The precision-recall curve shows the tradeoff between precision and recall for different threshold. A high Area Under Precision-Recall curve (AUPR) represents both high recall and high precision, show that the classifier is returning accurate results (high precision), as well as returning many of all positive results (high recall). It ranges between 0 and 1.
•***AUROC***: The Area Under the Receiver Operating Characteristic (AUROC) curve is another frequently used metric to describe the goodness of a binary classifier. It is equal to the probability that the predictor will rank a randomly chosen positive sample higher than a randomly chosen negative sample. Usually, it ranges between 0.5 and 1, where 0.5 means the classifier performs no better than random guesses, and 1 if the classifier perfectly separates positive samples from negative.

#### 2.3.3 Descriptor construction

There have been many developed descriptors for enzymes and chemicals available [28,33]. The current pipeline constructs the descriptors in a modular style and introduces descriptors for enzymes and chemicals separately. It is illustrated in Supplementary Method S2. Here are the modules used:

For enzymes,

• ***iFeature***: *iFeature* is a comprehensive Python-based toolkit for generating various numerical feature representation schemes from protein or peptide sequence [34]. Here it is used to calculate sequence-based descriptors for enzymes to incorporate the physicochemical properties and statistically relevant quantities. The specific features selected and their meaning is provided in Supplementary Method S3.
• ***ESM-2***: *Evolutionary Scale Modeling* (*esm*) is the general-purpose pretrained language model for proteins developed by Meta [35,36]. It was trained on 250 million protein sequences and outperforms all tested single-sequence protein language models in a series of structure prediction tasks. Here it is used as a representation of deep-learning-based descriptors from protein sequence. The model used is *esm2_t33_650M_UR50D.*
• ***Protdcal***: *ProtDCal* is a user-friendly java-based software package that was developed to generate a variety of numeric descriptors for protein structures and sequences [37]. Here it is used to calculate the stability for enzymes using the predicted structures. The template Protdcal project file is provided in Supplementary Method S4.
• ***Swiss-Model***: *Swiss-Model* is a fully automated protein structure homology-modelling server [38]. Here the web server is used for homology-modelling structure prediction. The quality of predicted structures is assessed by the QMEAN Z-score [39].
• ***ESM-Fold***: *ESM-Fold* was developed by meta to quickly predict the structure of a single sequence based on the esm model. It is much faster than alphafold2 but has comparable accuracy [36]. Here it is used for *de novo* structure prediction. The model used is *esmfold_3B_v1.* The quality of predicted structures is assessed by the pLDDT score [40].

For chemicals.

• ***Rcpi***: it is used to calculate the physicochemical properties from the 2D and 3D structure of the chemicals [41]. The R scripts is provided in Supplementary Method S5.

To build the benchmark model, two randomization approaches were used to generate descriptors based on the constructed pipelines (Supplementary Method S2).

• ***shuffle***: The shuffle strategy randomly rearranges the amino acids that constitute a protein sequence, so that the original sequence order information and relative position information are no longer maintained.
• ***permutation***: The permutation strategy is used for a calculated descriptor of the enzymes or chemicals in the data set to randomly recombine the objects and the calculated descriptors so that the original correspondences are no longer maintained.

#### 2.3.4 Machine learning model

The concatenation of the two numerical descriptors for the enzyme and the chemical is as the input of the machine learning model. Then a predicted number is as the output for evaluation. For regression problems, it is the fitted value of the activity data; for classification problems, it is an activity probability value distributed between 0 and 1. Values greater than a certain activity threshold are active.

Though there have been a variety of machine learning models which could be conveniently implemented using modern frameworks, we mainly use the Random Forest model as the workhorse, because it has been reported as the best one in previous work for such a task [26,27] and is suitable for current dataset size [42]. In addition, using the same model architecture could already achieve the main purpose of the study to compare different descriptor strategies.

Four types of Random Forest models are constructed depending on whether a randomization strategy is used to corrupt information.

• ***RF with all descriptors***: Random Forest model with all descriptors uses the regular pipeline with no shuffling or permutation strategy.
• ***RF with protein descriptor***: For Random Forest model with protein descriptor, after the regular pipeline to build the descriptors, use the permutation strategy for chemical descriptors. Since the correspondence between the chemicals' labels and the descriptors is destroyed, it is believed that the model only remembers which chemical is in the training dataset but could not actually use the chemical information. So, it is a machine learning model that only contains protein descriptor but not chemical descriptor.
• ***RF with chemical descriptor***: For Random Forest model with chemical descriptor, after the regular pipeline to build the descriptors, use the permutation strategy for protein descriptors. In the same way as RF with protein descriptor, the model cannot generalize to new proteins due to permutated protein labels.
• ***RF with no descriptor***: For Random Forest model with no descriptor, after the pipeline to build the descriptors, both descriptors for chemicals and proteins are permutated. Then the Random Forest model has no useful information for proteins or chemicals.

The last three types of models above serve as benchmark models to indicate whether introducing descriptors improves model performance. *ifeature* is used as the default protein descriptor and *Rcpi* as the default chemical descriptor.

Machine learning models were constructed using scikit-learn (version 1.2.0). And the hyperparameters were optimized using optuna (3.1.0) with three parallel experiments for ten-fold cross-validation. For classification, the optimization objective is maximizing the precision. For regression, it is minimizing the mean absolute error.

#### 2.3.5 Benchmark models

For new enzyme evaluation (LPOT) and new chemical evaluation (LCOT), two additional baseline models were constructed without using the machine learning framework.

• ***random model***: The random model does not use any chemical or enzyme information, but only generates a predicted activity probability from a random distribution. It takes the activity ratio of the training set as the only parameter for the random distribution, whose value range is between 0 and 1. The random distribution could generate the activity probability for the input combination of chemical and enzyme. If it is greater than 0.5, the combination is predicted as active.
• ***activity model***: The activity model does not use any chemical or enzyme descriptor. For new enzyme evaluation (LPOT), activity predictions are performed on known chemicals. The activity model uses the training set data to rank these chemicals based on their total activity on all known enzymes. For any new enzyme, the top-*k* chemicals ranked in the training dataset are predicted as active, while the others are inactive. *k* equals the number of chemicals times the activity ratio of the training dataset.

An illustration of the two models is given in the Supplementary Method S6. Other published models as benchmarks which also predict the enzyme chemical relationship are described in the Supplementary Table S3-S5.

## 3 Results and discussion

### 3.1 Evaluations of new relationships

**Fig. 3:**
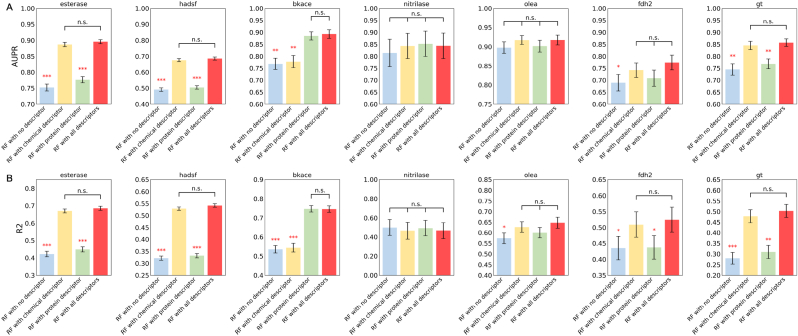
**(A) AUPR for the classification and (B) R2 for the regression on seven datasets of new relationship evaluation (LOOT).** Standard error bars indicate the standard error of the mean of metrics computed for 10-fold cross-validations on 5 splits controlled by random seeds. Each model is compared to the Random Forest model with all descriptors (RF with all descriptors) using a standard independent two sample test. The asterisks account for the significance level: * for 0.05, ** for 0.01, *** for 0.001. Each dataset has its own y-axis to emphasize differences between models.

We initially validated the machine learning pipeline and studied the impact of various descriptors in the new enzyme-substrate relationship prediction problems. As illustrated in Fig. 3, the Random Forest model with all descriptors results in the best metrics with statistically significant for both the classification and regression problems for 5 out of the 7 datasets. It can also be seen that models perform differently on different datasets after introducing protein or chemical descriptors. For esterase, hadsf and gt, the incorporation of chemical descriptors notably improves the performance. And for bkace, the model mainly benefitted from the protein descriptors. And for nitrilase, olea and fdh2 datasets, neither the chemical nor protein descriptors significantly impacted the predictions. The results could be explained by the number of the chemicals and enzymes. As shown in Fig. 1 and Supplementary Table S1, esterase, hadsf and gt datasets have the most chemicals, while bkace has the highest ratio of enzymes to chemicals, so in these two types of datasets, chemical descriptors and enzyme descriptors play a dominant role respectively. For the three smaller datasets, the predictions were more affected by the activity data themselves, and the descriptors of the chemicals or enzymes did not seem to play a decisive role. Notably, the absolute performance of the model for classification problem on these three datasets is good enough, which challenges the need to develop descriptors for small-scale projects to discover the initial enzymatic activity.

As shown in Fig. 3B, the current model performs poorly on quantitative prediction, with R2 values ranging from 0.45 to 0.75. The regression problem is similar to the prediction of kcat values targeted by DLkcat [43], where kcat is also a continuous number. We use the default DLKcat model for regression (Supplementary Table S3). The obtained R2 is close to 0, like a random prediction. When activity quantification is used to estimate activity values for combinations of enzymes and chemicals that are known to be active, the model performance remains unsatisfactory. For the hadsf and fdh2 data sets, R2 is lower than 0.5 and for the nitrilase, R2 is close to 0 (Supplementary Fig. S2C). It is believed that accurately quantify activity for the enzymes as the whole cell or crude enzyme preparation is difficult due to the interference of other factors. However, the status of the enzyme is not a factor that affects the performance of the regression models (Supplementary Table S1). Quantitative prediction of enzyme activity remains a difficult problem [26].

### 3.2 Evaluations of new enzymes

Evaluations of new enzymes *in silico* is necessary to reduce the cost and timeline for new biocatalytic process [44]. As shown in Fig. 1, The average top-5 similarity of enzymes of the datasets ranges from 26.5% (esterase) to 64.8% (bkace), which could represent the application scenario of mining function-related enzymes from genome databases.

**Fig. 4:**
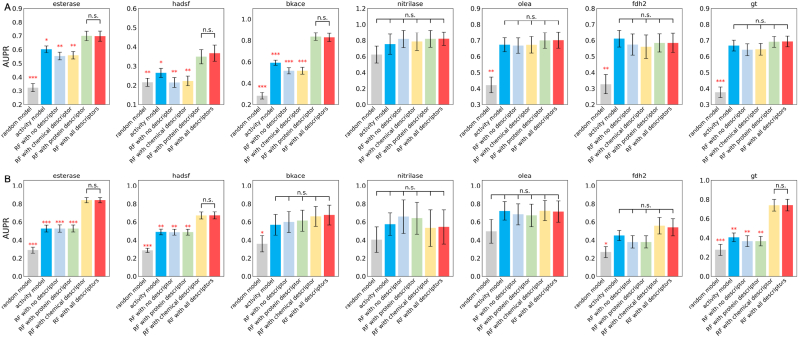
**AUPR for the classification on seven datasets of (A) new enzyme evaluation (LPOT) and (B) new chemical evaluation (LCOT).** Standard error bars indicate the standard error of the mean of metrics computed for 10-fold cross-validations on 5 splits controlled by random seeds. Each model is compared to the Random Forest model with all descriptors (RF with all descriptors) using a standard independent two sample test. The asterisks account for the significance level: * for 0.05, ** for 0.01, *** for 0.001. Each dataset has its own y-axis to emphasize differences between models.

Fig. 4A shows the performance of Random Forest models on all seven datasets. To clarify the role of descriptors, we constructed two non-machine learning models as baselines. Compared to the random model (gray), RF with all descriptors model (red) demonstrated considerable improvements. When using the activity model (navy blue) as the benchmark, the addition of protein and chemical descriptors did not improve model performance for the four smaller datasets. And protein descriptors could improve the model for the three larger datasets (esterase, hadsf, and bkace). The activity model (navy blue) is marginally stronger than the RF with no descriptor model (light blue), which employed a permutation method to eliminate biological and chemical information. This could be because the model's knowledge of enzyme connections is hampered by the ambiguous representation. Another discovery worth noting is that the introduction of chemical descriptors did not increase the model's performance, as evidenced by the comparison of RF with all descriptors model (red) and RF with protein descriptor model (green), as well as the comparison of RF with chemical descriptor model (yellow) and RF with no descriptor model (blue). This observation is consistent with the work of Goldman et al. [29], who demonstrated that introducing additional chemical information does not necessarily increase the accuracy of predictions of new enzyme activity on known chemicals. The findings emphasize the importance of protein descriptors in enzyme research. Similar observations might be made with the AUROC as the metric (Fig. S3A).

### 3.3 Evaluations of new chemicals

Enzyme engineering also aims to broaden the substrate range of developed enzymes [45]. The means of top-5 substrate similarity of available datasets range from 26.4% (fdh2) to 63.8% (olea), indicating the typical cases of substrate profile screening from commercial libraries or metabolic pathways.

Fig. 4B shows that introducing chemical descriptors improved the performance of RF models for esterase, hadsf, fdh2 and gt datasets while having little effect on the bkace, nitrilase and olea datasets. Perhaps this is still due to the difference in data size, as the first four datasets contain the most chemicals. The observation, like that for LPOT, was that incorporating protein descriptors into the RF model did not improve predictions. This highlights the significance of chemical descriptors in predicting the activity of new chemicals to known enzymes.

### 3.4 Comparison of different descriptors for protein

**Fig. 5:**
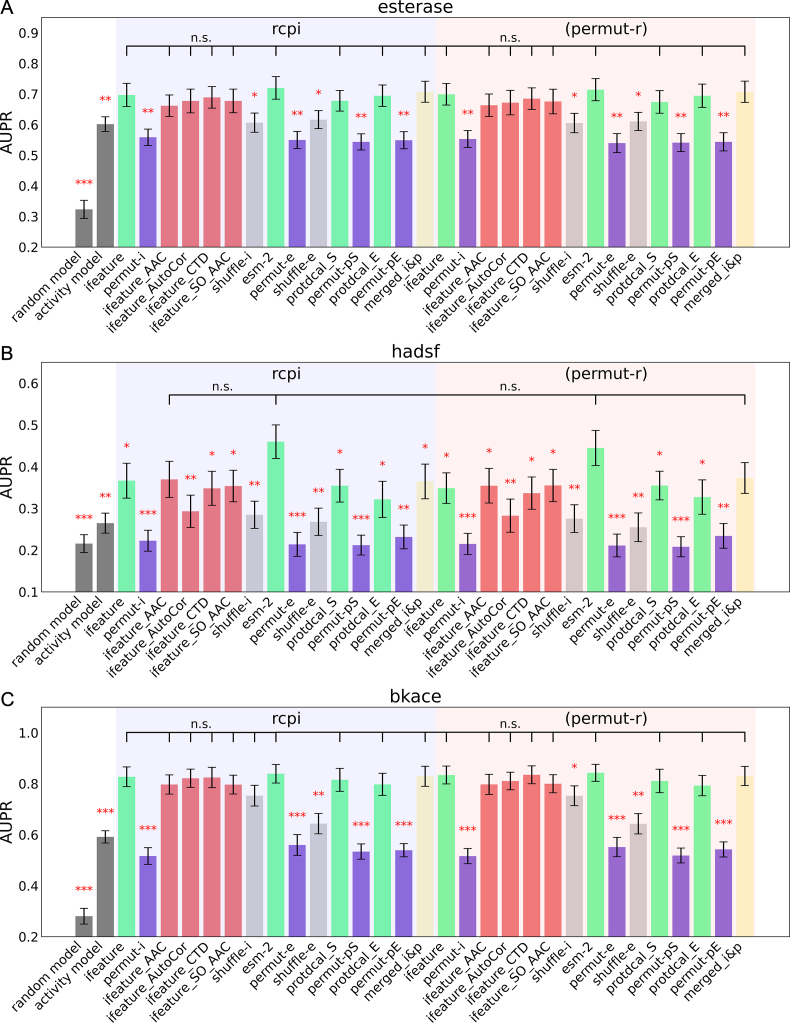
**AUPR for the classification of new enzyme evaluation (LPOT) on (A) esterase, (B) hadsf and (C) bkace dataset.** Machine learning models with different descriptor combinations and two baseline models (random model and activity model) are compared. Chemical descriptors are rcpi or its permutation one (permut-r). A total of 15 protein descriptors were used. The green four (ifeature, esm-2, protdcal-E, protdcal-S) use different module to calculate the descriptors and the purple four (permut-i, permut-e, permut-pE, permut-pS) are their permutation version. The light gray ones (shuffle-i, shuffle-e) are the shuffle version of the two sequence-based descriptors. The red four (ifeature_AAC, ifeature_AutoCor, ifeature_CTD, ifeature_SO_AAC) are taken disjoint subsets from ifeature descriptors. The yellow one (merged_i&p) is the descriptor that combine ifeature and protdcal_S descriptors. For the pipeline of all 15 descriptors, refer to Supplementary Method S2. Standard error bars indicate the standard error of the mean of metrics computed for 10-fold cross-validations on 5 splits controlled by random seeds. Each model is compared to the Random Forest model with descriptors (esm-2, rcpi) using a standard independent two sample test. The asterisks account for the significance level: * for 0.05, ** for 0.01, *** for 0.001. Each dataset has its own y-axis to emphasize differences between models.

The importance of protein descriptors is demonstrated by the evaluation of new enzymes. Using the present modular framework, we conducted a thorough comparison of several enzyme embedding strategies. Fig. 5 depicts the performance of RF models with different protein and chemical descriptor combinations on the esterase, hadsf, and bkace datasets, where protein descriptors could significantly enhance performance. Among all combinations, the model with esm-2 as the protein descriptors produced the best results, particularly on the hadsf dataset, where it outperformed the calculation-based ifeature and protdcal descriptors. This demonstrates the enormous power of deep learning-based embeddings in predicting protein function [24,46].

Two randomization approaches are used to eliminate the protein information for descriptors based on amino acid sequence. The primary structure of the enzyme is destroyed by the shuffling strategy, resulting in a considerable drop in model performance (as illustrated by comparisons of ifeature with shuffle-i, and by esm-2 with shuffle-e). However, it is still better than the permutation ones (compare shuffle-i with permut-i and shuffle-e with permut-e), suggesting that the protein's composition still encode some of the functional information.

The ifeature descriptor is created by picking some of the descriptors from the ifeature implementations (Supplementary Method S3). Its disjoint subsets are used to investigate roles of these descriptors. The performances of the four subset ifeature descriptors were equivalent to that of the complete ifeature descriptor in most cases, showing that these subset descriptors encode similar primary structure information of the protein. It is worth noticing that the ifeature_AAC descriptor performs just slightly worse than the full 1233-dimensional descriptor in all three datasets. The former is a 50-dimensional vector that includes the amino acid composition (AAC, 20 dimensions), grouped amino acid composition (GAAC, 5 dimensions), and grouped dipeptide composition (GDPC, 25 dimensions) of the protein. This could imply that most of the information for artificially constructed descriptors is already present in the low-level protein compositions. The capacity of deep learning algorithms to capture higher-order correlations in protein sequences may explain why they could outperform those artificially constructed descriptors [47,48].

ProtDCal [37] was used to create structure-based descriptors, which calculates the overall stability of the folded structure (Supplementary Method S4). The performance difference between models employing protdcal or ifeature descriptors is not significant. The ifeature&protdcal descriptor created by fusing the two descriptors likewise failed to increase performance further. This could imply that the current approach of embedding protein structure is too naive to provide additional information to sequence descriptors. The performance of the protdcal descriptor is also independent of the structure prediction strategy.

Comparing the best results that we obtained (RF with esm-2 and Rcpi descriptors) to those by CPI model [29] shows that our RF-based results outperform those based on MLP (Supplementary Table S4). It could reflect the benefits of traditional machine learning methods in small data problems like protein family function prediction. Predictions using the ES_pred model produced lower AUPR values when not retrained on each dataset (Supplementary Table S5). This confirms the importance to retrain general models on specific datasets [30].

Furthermore, some interesting cases were discovered in the evaluation in the classification problem of new relationships (Fig. S6). As depicted in Fig. 5B, esm-2 showed significantly better performance on the new enzyme evaluation problem on the hadsf dataset. However, on the LOOT problem on the same data set, the structure-based ProtDcal descriptor showed better performance (Fig. S6B). This suggests that the descriptor strategies for enzyme design should be carefully studied for different application scenarios.

### 3.5 Visualization of enzymes in different feature space

**Fig. 6:**
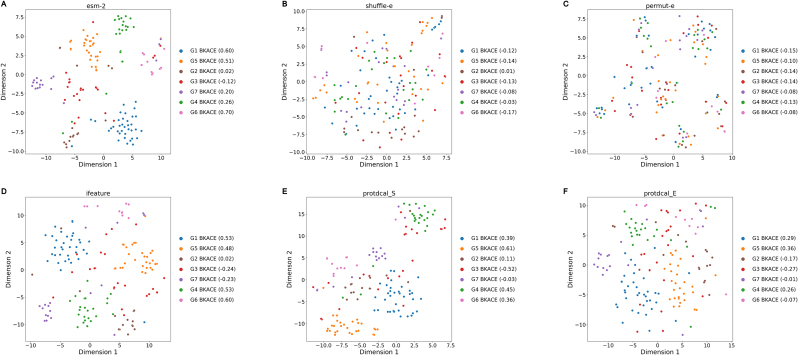
**The results of t-SNE dimension reduction for enzymes of the bkace dataset using (A) esm-2, (B) shuffle-e, (C) permut-e, (D) ifeature, (E) protdcal_S, (F) protdcal_E as enzyme descriptors, respectively.** The activity-based enzyme family in the original literature was used as the clustering label. The clustering coefficient was calculated for each cluster, which takes a value from −1 to 1. A value of 0 indicates random clustering. The closer it is to 1, the more distinct the cluster is from others.

The observed differences in model performance across protein descriptors prompted investigations into their ability to convey information about enzyme function. The nonlinear dimensionality reduction strategy, t-SNE was used to view enzymes in feature spaces for different descriptors. The introduction of protein descriptors improved model performance for LPOT in the bkace dataset significantly. As shown in Fig. 6, when using functional families as clustering labels, esm-2, ifeature and protdcal-E descriptors could cluster all the families well, as evidenced by the clustering coefficients and visual inspection. Conversely, the clustering coefficients obtained for shuffle-e and permut-e are close to zero, indicating that both randomization strategies destroy the functional correlation between proteins which were captured by the primary structure of the enzymes and the descriptors.

Similar clustering behaviors were observed for the esterase dataset, such as for FIV, C-C MCPh, FVII and other superfamilies (Fig. S7). But for the hadsf dataset, the clustering based on different protein cap domains were not obvious (Fig. S8), indicating a more complex “sequence-structure-function” relationship of the hadsf family. This may partly explain the poor performance of machine learning models in the latter.

### 3.6 Correlation of performance of models with statistics of datasets

We have proved that the descriptor strategy could be more beneficial for datasets containing enough enzymes and chemicals, as the descriptors could capture the complex interaction patterns presented in such datasets. Another issue is the applicability of the machine learning models themselves. To emphasize the features of the dataset to which machine learning models are suitable, we correlated the statistical properties of the dataset to the model's metrics on the evaluation problem. The correlation coefficients between the statistics were first computed (Supplementary Fig. S13A). The correlation between the number of enzymes, top-5 protein similarity and activity order index at enzyme axis (aoie) is negative, which means that the larger the dataset, the less similarity and the more specificity proteins are. Top-5 protein similarity and aoie have a low correlation, showing that they are independent parameters in characterizing the enzyme properties of the dataset. The number of chemicals, top-5 chemical similarity, and activity order index at chemical axis (aoic) are not highly correlated, indicating that they could be used independently for properties of the dataset related to chemicals.

**Fig. 7:**
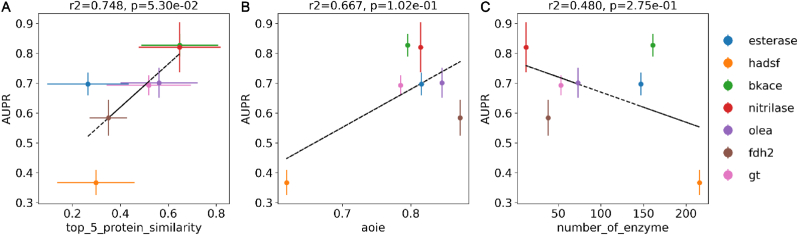
**The correlation of the AUPR metric on LPOT problem of RF with all descriptors against (A) the top-5 protein similarity, (B) aoie and (C) the number of enzymes. Each point represents a dataset.** The vertical error bar shows the standard deviation of the model predictions. The horizontal error bar shows the standard deviation of the similarity data. A linear regression was attempted, which is indicated by the black dashed line. The correlation coefficients (r2) and significance values (p-values) were calculated.

The models' LPOT performance were then correlated with the dataset's statistics. The AUPR of the RF model corresponded most significantly with the top-5 sequence similarity of the enzymes, as shown in Fig. 7A. The finding was consistent with deep learning models [29]. The relationship between aoie and LPOT's AUPR revealed that predictions are more challenging for enzyme family with lower or higher specificity, for example, the fdh2 and hadsf dataset (Fig. 7B). The number of enzymes and activity ratio had a significant impact on the performance of the baseline model, e.g. on the dataset whose size was small and whose activity ratio was high, the activity model already performed well, and the introduction of protein descriptors had little impact (Fig. S13B). Conversely, the hadsf dataset had the greatest number of enzymes, which might be a challenge for the machine learning models (Fig. 7C).

Model performance for new chemical evaluations was also connected with dataset statistics. Fig. S15E demonstrated a substantial association between model performance and the top-5 chemical similarity. The performance of LCOT's AUPR is weakly correlated with the number of compounds and aoic (Figs. S15F and S15G). Interestingly, the RF model's LCOT performance is positively correlated with the number of enzymes (Fig. S15C), whereas the LPOT performance of the RF model is negatively correlated with the number of chemicals (Supplementary Fig. S14D). It might suggest that we could consider the enzymes and chemicals in an asymmetric way.

## 4 Conclusions

Predicting enzyme-chemical relationships precisely is critical to accelerating the pace of new enzyme discovery. Considering the complexity of enzyme catalysis, diverse descriptor strategies were integrated into the Random Forest model. Our study retrospectively evaluates the impact of these descriptors on the prediction of enzyme-substrate relationships using high-quality experimental datasets assayed under consistent conditions. In three evaluation settings, we discovered the varied roles of the protein and chemical descriptors. The consensus conclusion is that the employment of descriptor strategies in activity screening efforts against genomic databases is expected to achieve performance over baseline models only when the dataset contains a substantial number of enzymes or chemicals. This is critical in protein engineering. The advantage is that researchers could use the small amount of activity data at hand to quickly build a simple model to guide initial enzyme discovery attempts, such as the activity model in this work or the k-nearest neighbor model. A comparison of multiple protein descriptors revealed that both human-made and deep-learning-based descriptors could improve the model's performance on the new enzyme evaluation task and the latter appeared to be more promising. The visualization of enzymes by t-SNE in the feature space demonstrated the capacity of the esm-2, ifeature and protdcal descriptors to capture functional relevance of enzymes. The attempt to correlate the model performance and dataset statistics showed that assessing model generalization remained an open problem. Further study should focus on enhancing pretrained protein sequence language models by incorporating information of the protein structure that is complementary to the sequence information, such as descriptors describing enzyme catalytic motifs or catalytic pockets. Advances in descriptor strategies are expected to infuse our understanding of enzymatic catalytic processes into data-driven modelling to improve the utility of enzymes in chemical synthesis.

## CRediT authorship contribution statement

**Yilei Han:** Conceptualization, Methodology, Software, Data curation, Writing – original draft. **Haoye Zhang:** Software, Data curation. **Zheni Zeng:** Software, Data curation. **Zhiyuan Liu:** Supervision, Software, Methodology. **Diannan Lu:** Supervision, Writing – review & editing. **Zheng Liu:** Conceptualization, Writing – review & editing.

## Declaration of competing interest

The authors declare that they have no known competing financial interests or personal relationships that could have appeared to influence the work reported in this paper.
