# Supplementary material for: Descriptor-augmented machine learning for enzyme-chemical interaction predictions
Source: Synth Syst Biotechnol. 2024 Feb 28;9(2):259–68. doi: 10.1016/j.synbio.2024.02.006 (PMC10915406; doi:10.1016/j.synbio.2024.02.006)
Supplement: Supplementary Methods, Tables and Figures [file mmc1.docx]

**Supplementary**

[Method S1. Statistics of the datasets 2](#_Toc156511626)

[Method S2. Descriptor construction modules 3](#_Toc156511627)

[Method S3. ifeature descriptor list 4](#_Toc156511628)

[Method S4. ProtDCal project file 5](#_Toc156511629)

[Method S5. Rcpi R script 6](#_Toc156511630)

[Method S6. Activity model and random model 8](#_Toc156511631)

[Table S1. Metadata of the datasets 9](#_Toc156511632)

[Table S2. Statistics of the datasets 9](#_Toc156511633)

[Table S3. R2 for DLkcat model on LOOT 10](#_Toc156511634)

[Table S4. AUPR and AUROC for CPI model on LPOT and LCOT 10](#_Toc156511635)

[Table S5. AUPR for ES_pred model on new chemicals and new enzymes 11](#_Toc156511636)

[Figure S1. Top-5 Similarity distributions of proteins and chemicals 12](#_Toc156511637)

[Figure S2. Metrics for predictions on 7 datasets of LOOT 13](#_Toc156511638)

[Figure S3. AUROC for classification on 7 datasets of (A) LPOT and (B) LCOT 13](#_Toc156511639)

[Figure S4. AUPR for the classification of new enzyme evaluation (LPOT) 14](#_Toc156511640)

[Figure S5 AUPR for the classification of new chemical evaluation (LCOT) 15](#_Toc156511641)

[Figure S6 AUPR for the classification of new relation evaluation (LOOT) 17](#_Toc156511642)

[Figure S7. The results of t-SNE for the esterase dataset 19](#_Toc156511643)

[Figure S8. The results of t-SNE for the hadsf dataset 19](#_Toc156511644)

[Figure S9. The results of t-SNE dimension reduction for the nitrilase dataset 19](#_Toc156511645)

[Figure S10. The results of t-SNE dimension reduction for the olea dataset 20](#_Toc156511646)

[Figure S11. The results of t-SNE dimension reduction for the fdh2 dataset 20](#_Toc156511647)

[Figure S12. The results of t-SNE dimension reduction for the gt dataset 20](#_Toc156511648)

[Figure S13. Correlation coefficients (A) between statistics of datasets; (B) LPOT-AUPR against statistics of datasets; (C) LCOT-AUPR against statistics of datasets 21](#_Toc156511649)

[Figure S14. The correlation of the LPOT-AUPR of RF with all descriptors against statistics of datasets 21](#_Toc156511650)

[Figure S15. The correlation of the LCOT-AUPR of RF with all descriptors against statistics of datasets 21](#_Toc156511651)

[Figure S16. The distribution of pLDDT scores of ESM-fold *de novo* structure predictions for 7 datasets 22](#_Toc156511652)

[Figure S17. The distribution of QMEAN Z-scores of Swiss-model templated-based structure predictions for 7 datasets 22](#_Toc156511653)

[Reference 23](#_Toc156511654)

# Method S1. Statistics of the datasets

## S1.1 Activity Data Matrix and the activity ratio

Suppose there are *m* chemicals and *n* enzymes in one dataset. The activity data matrix is $A_{nm}$, which is either a 0-1 matrix, i.e., $a_{ij}\in\left[ 0 , 1 \right]$ or a continuous value matrix, i.e., $a_{ij}\in R^{*}$. The matrix element $a_{ij}$represents the activity between the ith enzyme and the jth chemical.

The activity ratio is defined by the following equation, which represents the average activity of the dataset:

$$ActivityRatio=\frac{\sum_{i}^{n} \sum_{j}^{m} a_{ij}}{n*m}$$

## S1.2 The promiscuity factors

The Activity Order Index at Enzyme axis (AOIE) defined for the dataset was calculated as follows.

First, the total activity of the chemicals is calculated:

$$sum\left( a \right)_{j}=\Sigma_{i}^{n}a_{ij}$$

Second, the counts of common chemical pairs (Common Pairs) and critical chemical pairs (Critical Pairs) on enzyme i are calculated:

$$\#common\left( a \right)_{i}=\Sigma_{j}^{m}\Sigma_{j^{'}}^{m}1(a_{ij}>a_{ij^{'}}) \& 1({sum(a)}_{j}>sum\left( a \right)_{j^{'}})$$

$$\#critical\left( a \right)_{i}=\Sigma_{j}^{m}\Sigma_{j^{'}}^{m}1(a_{ij}>a_{ij^{'}}) \& 1\left( sum\left( a \right)_{j}\leq sum\left( a \right)_{j^{'}} \right)$$

Aggregate the entire dataset to get the AOIE for the whole dataset:

$$AOIE=\frac{\Sigma_{i}^{n}\#common\left( a \right)_{i}}{\Sigma_{i}^{n}\#common\left( a \right)_{i}+\Sigma_{i}^{n}\#critical\left( a \right)_{i}}$$

Similarly, the Activity Order Index at Chemical axis (AOIC) can be calculated for the dataset:

First, the total enzyme activity is calculated as:

$$sum\left( a \right)_{i}=\Sigma_{j}^{m}a_{ij}$$

Second, the counts of common chemical pairs (Common Pairs) and critical chemical pairs (Critical Pairs) on chemical j are calculated:

$$\#common\left( a \right)_{j}=\Sigma_{i}^{n}\Sigma_{i^{'}}^{n}(a_{ij}>a_{i^{'}j})\&(sum\left( a \right)_{i}>sum\left( a \right)_{i^{'}})$$

$$\#critical\left( a \right)_{j}=\Sigma_{i}^{n}\Sigma_{i^{'}}^{n}(a_{ij}>a_{i^{'}j})\&\left( sum\left( a \right)_{i}\leq sum\left( a \right)_{i^{'}} \right)$$

Aggregate the entire dataset to get the AOIC for the whole dataset:

$$AOIC=\frac{\Sigma_{j}^{m}\#common\left( a \right)_{j}}{\Sigma_{j}^{m}\#common\left( a \right)_{j}+\Sigma_{j}^{m}\#critical\left( a \right)_{j}}$$

## S1.3 The top-5 chemical similarity and the top-5 sequence similarity

The top-5 sequence similarity and top-5 chemical similarity were calculated for each dataset referring to the definition of similarity in the literature.

Sequence similarity was calculated using the Smith-Waterman algorithm implemented in the Biopython package, aiming at local similarity of sequences.

Chemical similarity was calculated using the Tanimoto similarity of chiral 2048-bit Morgan fingerprints implemented in the RDKit package.

# Method S2. Descriptor construction modules


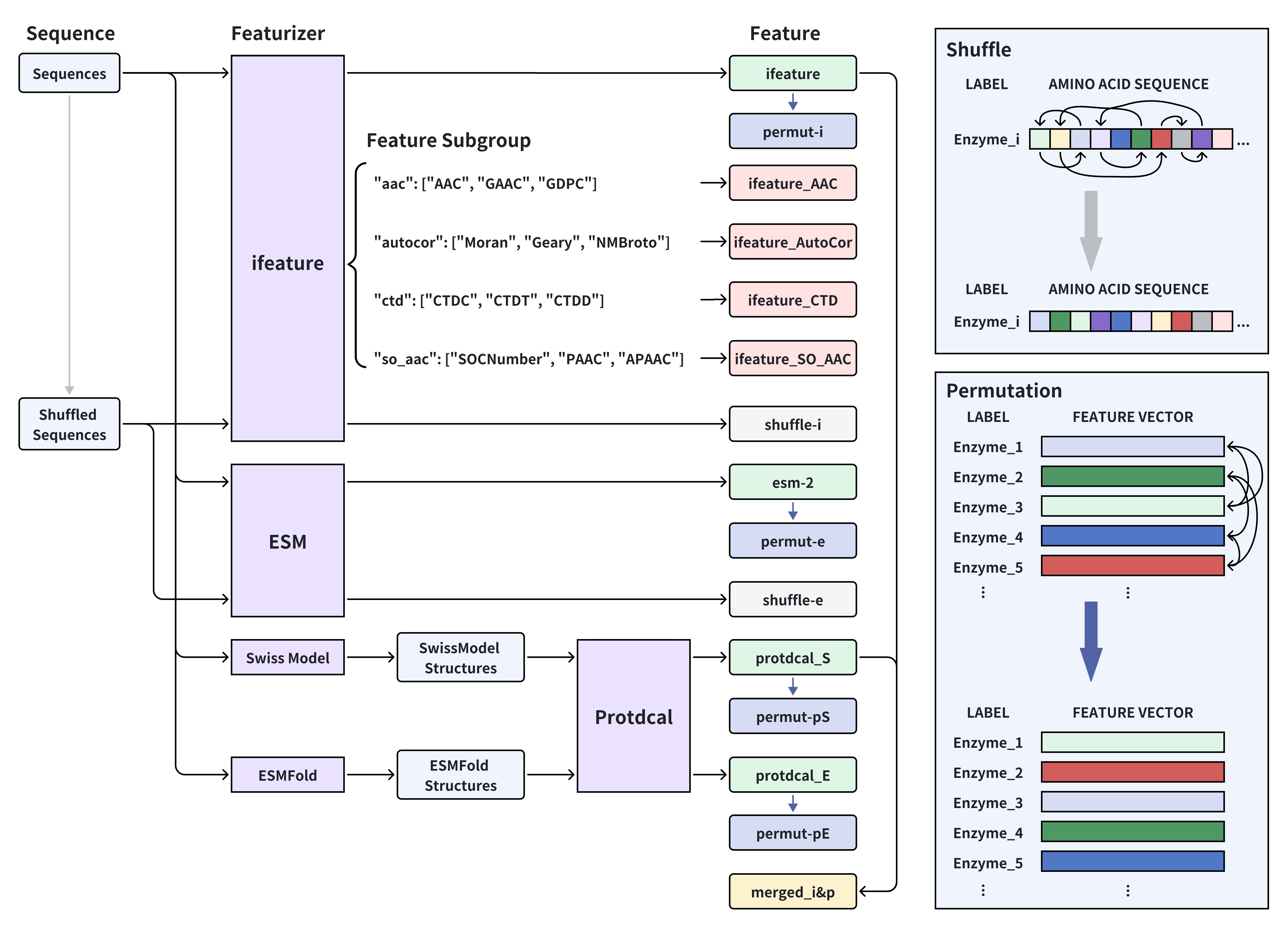


# Method S3. ifeature descriptor list

| **Group** | **Descriptor** | **Name** | **Dimension** | **Class** |
| --- | --- | --- | --- | --- |
| AAC | AAC | Amino acid composition | 20 | 1-mer(base) |
|  | GAAC | Grouped amino acid composition | 5 | 1-mer, grouped |
|  | GDPC | Grouped dipeptide composition | 25 | 2-mer, grouped |
| AutoCor | Moran | Moran | 240 | 2-mer, correlation |
|  | Geary | Geary | 240 | 2-mer, correlation |
|  | NMBroto | Normalized Moreau-Broto | 240 | 2-mer, correlation |
| CTD | CTDC | Composition | 39 | 1-mer, grouped |
|  | CTDT | Transition | 39 | 2-mer, grouped |
|  | CTDD | Distribution | 195 | distribution |
| SO_AAC | SOCNumber | Sequence-order-coupling number | 60 | 2-mer, k-gap, nnm |
|  | PAAC | Pseudo-amino acid composition | 50 | 2-mer, normed, sub |
|  | APAAC | Amphiphilic PAAC | 80 | 2-mer, normed, product |
|  | Total |  | 1233 |  |

# Method S4. ProtDCal project file

directory:
1_EH147/Datasets
indices:
DGc(F),Gw(F),Gs(F),W(F),HBd,DGs,DGw,DGel,DGLJ,DGtor,Gs(U),Gw(U),W(U),A,DA,DAnp,lnFD,Mw,HP,Pa,Z1,DHf,IP,Pb,Z2,Xi,ECI,ISA,Pt,Z3,L1-9,wR2,wFLC,wNLC,wNc,wSp,wLCO,wCTP,wCLQ
wR2:
Xi,DHf,ECI,IP,ISA,Mw,None,NumAtoms,TopDist,L1-9,Z1,Z2,Z3,HP,Ap
wFLC:
Xi,DHf,ECI,IP,ISA,Mw,None,NumAtoms,TopDist,L1-9,Z1,Z2,Z3,HP,Ap
wNLC:
Xi,DHf,ECI,IP,ISA,Mw,None,NumAtoms,TopDist,L1-9,Z1,Z2,Z3,HP,Ap
wNc:
Xi,DHf,ECI,IP,ISA,Mw,None,NumAtoms,TopDist,L1-9,Z1,Z2,Z3,HP,Ap
wSp:
Xi,DHf,ECI,IP,ISA,Mw,None,NumAtoms,TopDist,L1-9,Z1,Z2,Z3,HP,Ap
wLCO:
Xi,DHf,ECI,IP,ISA,Mw,None,NumAtoms,TopDist,L1-9,Z1,Z2,Z3,HP,Ap
wCTP:
Xi,DHf,ECI,IP,ISA,Mw,None,NumAtoms,TopDist,L1-9,Z1,Z2,Z3,HP,Ap
wCLQ:
Xi,DHf,ECI,IP,ISA,Mw,None,NumAtoms,TopDist,L1-9,Z1,Z2,Z3,HP,Ap
groups:
PRT
invariants:
P2,DE,SI,M,G
parameters(t_cont,s_cont,A%,HydGroup,n,bins,K,SubG):
4.0,8.0,5.0,9.4,3.0,50,5,3
options(decimals,harmonicMeanType,geometricMeanType,windexID,datasetType,outputOrder):
5,0,0,-1,pdb,true

# Method S5. Rcpi R script

library("Rcpi")
library("ChemmineR")

Args <- commandArgs()

sdffile <- Args[6]
smifile <- "smi_str.smi"
csvfile <- Args[7]

x.sdf = readMolFromSDF(sdffile)

sdfset <- read.SDFset(sdffile)
smiset <- sdf2smiles(sdfset)
write.SMI(smiset, smifile, cid=FALSE)

x.smi = readMolFromSmi(smifile, type = "mol")

x.df2 <- as.data.frame(matrix(nrow=1,ncol=0))

name_time <- data.frame(matrix(nrow=0,ncol=2))

for(a in c("extractDrugALOGP(x.sdf)",
 "extractDrugAminoAcidCount(x.sdf)",
 "extractDrugApol(x.sdf)",
 "extractDrugAromaticAtomsCount(x.sdf)",
 "extractDrugAromaticBondsCount(x.sdf)",
 "extractDrugAtomCount(x.sdf)",

 "extractDrugAutocorrelationPolarizability(x.sdf)",
 "extractDrugBCUT(x.sdf)",
 "extractDrugBondCount(x.sdf)",
 "extractDrugBPol(x.sdf)",
 "extractDrugCarbonTypes(x.sdf)",

 "extractDrugCPSA(x.sdf)",

 "extractDrugECI(x.sdf)",

 "extractDrugFMF(x.sdf)",
 "extractDrugFragmentComplexity(x.sdf)",

 "extractDrugGravitationalIndex(x.sdf)",
 "extractDrugHBondAcceptorCount(x.sdf)",
 "extractDrugHBondDonorCount(x.sdf)",

 "extractDrugHybridizationRatio(x.sdf)",

 "extractDrugKierHallSmarts(x.sdf)",

 "extractDrugLargestChain(x.sdf)",
 "extractDrugLargestPiSystem(x.sdf)",
 "extractDrugLengthOverBreadth(x.sdf)",
 "extractDrugLongestAliphaticChain(x.sdf)",

 "extractDrugMannholdLogP(x.sdf)",

 "extractDrugMomentOfInertia(x.sdf)",

 "extractDrugRotatableBondsCount(x.sdf)",
 "extractDrugRuleOfFive(x.sdf)",

 "extractDrugTPSA(x.sdf)",
 "extractDrugVABC(x.sdf)",
 "extractDrugVAdjMa(x.sdf)",
 "extractDrugWeight(x.sdf)",

 "extractDrugWHIM(x.sdf)",

 "extractDrugXLogP(x.sdf)",

 "extractDrugIPMolecularLearning(x.smi)",

 "extractDrugAutocorrelationCharge(x.smi)",
 "extractDrugAutocorrelationMass(x.smi)",
 "extractDrugChiChain(x.smi)",
 "extractDrugChiCluster(x.smi)",
 "extractDrugChiPath(x.smi)",
 "extractDrugChiPathCluster(x.smi)",

 "extractDrugKappaShapeIndices(x.smi)",

 "extractDrugMDE(x.smi)",

 "extractDrugPetitjeanNumber(x.smi)",
 "extractDrugPetitjeanShapeIndex(x.smi)",

 "extractDrugWeightedPath(x.smi)",

 "extractDrugWienerNumbers(x.smi)",

 "extractDrugZagrebIndex(x.smi)"
)
){
 t1 = proc.time()
 cat(a, "\n")
 f <- eval(parse(text = a))
 t2=proc.time()
 t=t2-t1
 cat(t[3][[1]], "\n")
 rbind(name_time, data.frame(Name=a, Time=t[3][[1]])) -> name_time
 cbind(x.df2, f) -> x.df2
}

write.csv(x.df2, csvfile, row.names = TRUE)
write.csv(name_time, "name_time.csv")

# Method S6. Activity model and random model


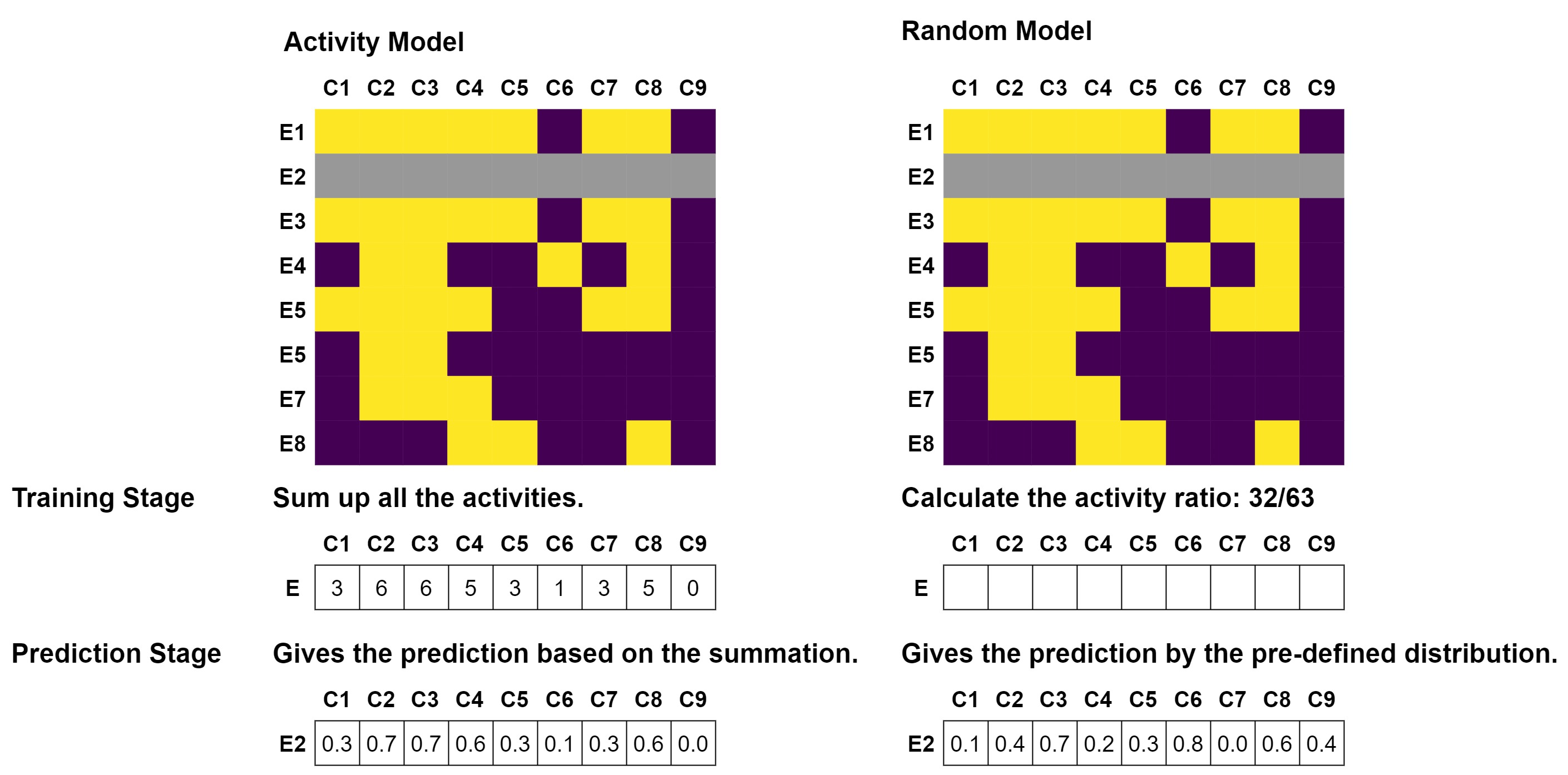


# Table S1. Metadata of the datasets

| **Dataset** | ***Type*** | ***Field*** | ***EC*** | ***#E*** | ***(#E)*** | ***#C*** | ***(#C)*** | ***#A*** | ***Ref*** |
| --- | --- | --- | --- | --- | --- | --- | --- | --- | --- |
| **esterase** | Cell | Organic | 3 | 147 | 107 | 96 | 58 | 14112 | [1] |
| **hadsf** | Pure | Metabolic | 3 | 216 | 100 | 167 | 99 | 36072 | [2] |
| **bkace** | Raw | Metabolic | 2 | 163 | 91 | 17 | 13 | 2771 | [3] |
| **nitrilase** | Raw | Organic | 3 | 12 | 12 | 20 | 14 | 240 | [4] |
| **olea** | Cell | Organic | 3 | 73 | 70 | 15 | 11 | 1095 | [5] |
| **fdh2** | Pure | Organic | 1 | 38 | 15 | 62 | 24 | 2356 | [6] |
| **gt** | Raw | Metabolic | 2 | 53 | 50 | 58 | 30 | 3074 | [7] |

Seven datasets are included for evaluation. Here are descriptions of the columns:

- *Type*: the status of the enzyme for activity assay. Cell: enzymes in the whole cell. Raw: the crude enzyme extract. Pure: purified enzyme.
- *Field*: the source of the substrate library. Organic: synthesized chemicals. Metabolic: endogenous metabolites of cells.
- *EC*: the enzymatic reaction type. EC1: oxidoreductase, EC2: transferase, EC3: hydrolase.
- *#E*: the number of enzymes.
- (#E): the number of enzymes in the new chemical evaluation (LCOT).
- *#C*: the number of chemicals.
- *(#C)*: the number of chemicals in the new enzyme evaluation (LPOT).
- *#A*: the number of active data points (number of enzymes * number of chemicals)
- *Ref*: the data source.

# Table S2. Statistics of the datasets

| **Dataset** | ***Activity Ratio*** | ***AOIE*** | ***AOIC*** | ***Top-5 protein similarity*** | ***Top-5 chemical similarity*** |
| --- | --- | --- | --- | --- | --- |
| **esterase** | 21.8% | 90.10% | 87.4% | 26.5%±17.0% | 57.1%±21.5% |
| **hadsf** | 14.8% | 75.8% | 84.9% | 29.8%±16.2% | 63.1%±16.0% |
| **bkace** | 22.0% | 84.5% | 88.5% | 64.8%±16.0% | 49.7%±13.2% |
| **nitrilase** | 47.1% | 88.0% | 83.2% | 64.7%±17.1% | 35.4%±12.3% |
| **olea** | 50.2% | 92.6% | 84.3% | 56.1%±16.1% | 63.8%±17.9% |
| **fdh2** | 13.2% | 96.3% | 90.6% | 35.0%±7.8% | 26.4%±11.2% |
| **gt** | 25.5% | 85.8% | 71.5% | 51.9%±17.7% | 38.8%±15.7% |

# Table S3. R2 for DLkcat model on LOOT

| **Datasets** | **esterase** | **hadsf** | **bkace** | **nitrilase** | **olea** | **fdh2** | **gt** |
| --- | --- | --- | --- | --- | --- | --- | --- |
| **dl-kcat** | -0.0038 | 0.0093 | -0.0329 | 0.0885 | -0.0571 | 0.0302 | -0.0582 |
| **Best RF**  **(protdcal_s, rcpi)** | 0.700±0.012 | 0.555±0.007 | 0.746±0.018 | 0.467±0.105 | 0.640±0.026 | 0.541±0.043 | 0.506±0.031 |

DLKcat is a deep learning model developed by Li et al. for predicting the enzymatic kinetic constant kcat to estimate the parameters of the Genome-scale Metabolic model [8]. It employed Graph Neural Network for chemicals and Convolutional Neural Networks for proteins. The DLKcat model is used predicted to predicted kcat values on all enzyme and substrate pairs in each dataset. Here it is used without re-train. The python script for DLkcat was used to perform the prediction of kcat values on the full dataset using amino acid sequences and chemical SMILES as input. R2 between the predicted kcat values and the true activity values was calculated on the all datasets.

# Table S4. AUPR and AUROC for CPI model on LPOT and LCOT

Goldman et al.[9] utilized esm-1b and JT-VAE to embed protein sequences and chemicals, respectively, in their deep learning models. We obtained the metric results from literature, where CPI model refers to the “FFN: [ESM-1b, Morgan]” model in the original article and best model refers to the model with the best metrics among the individual models in the original article. The correspondences between data sets are: esterase - Esterase, bkace - BKACE, fdh2 - Halogenase, hadsf - Phosphatase, olea - Thiolase, gt - Glyco.

**LPOT_AUPR**

| **Datasets** | **esterase** | **hadsf** | **bkace** | **nitrilase** | **olea** | **fdh2** | **gt** |
| --- | --- | --- | --- | --- | --- | --- | --- |
| **CPI Model** | 0.579±0.007 | 0.386±0.006 | 0.478±0.012 | - | 0.536±0.027 | 0.489±0.025 | 0.581±0.008 |
| **Best Model** | 0.588±0.011 | 0.413±0.005 | 0.664±0.011 | - | 0.552±0.013 | 0.510±0.043 | 0.581±0.008 |
| **Best RF**  **(esm-2, rcpi)** | 0.720±0.037 | 0.460±0.040 | 0.839±0.036 | 0.830±0.103 | 0.681±0.058 | 0.604±0.064 | 0.698±0.036 |

| **Datasets** | **esterase** | **hadsf** | **bkace** | **nitrilase** | **olea** | **fdh2** | **gt** |
| --- | --- | --- | --- | --- | --- | --- | --- |
| **CPI Model** | 0.723±0.003 | 0.676±0.008 | 0.793±0.001 | - | 0.637±0.025 | 0.568±0.036 | 0.636±0.019 |
| **Best Model** | 0.730±0.004 | 0.703±0.002 | 0.896±0.003 | - | 0.651±0.032 | 0.616±0.043 | 0.653±0.000 |
| **Best RF**  **(esm-2, rcpi)** | 0.824±0.020 | 0.719±0.024 | 0.929±0.019 | 0.857±0.047 | 0.741±0.040 | 0.754±0.049 | 0.786±0.022 |

**LPOT_AUROC**

| **Datasets** | **esterase** | **hadsf** | **bkace** | **nitrilase** | **olea** | **fdh2** | **gt** |
| --- | --- | --- | --- | --- | --- | --- | --- |
| **CPI Model** | 0.674±0.019 | 0.462±0.006 | - | - | - | 0.471±0.005 | 0.673±0.010 |
| **Best Model** | 0.841±0.003 | 0.506±0.004 | - | - | - | 0.525±0.000 | 0.699±0.008 |
| **Best RF**  **(ifeature, rcpi)** | 0.840±0.029 | 0.671±0.038 | 0.676±0.111 | 0.545±0.189 | 0.713±0.118 | 0.540±0.094 | 0.742±0.060 |

**LCOT_AUPR**

**LCOT_AUROC**

| **Datasets** | **esterase** | **hadsf** | **bkace** | **nitrilase** | **olea** | **fdh2** | **gt** |
| --- | --- | --- | --- | --- | --- | --- | --- |
| **CPI Model** | 0.808±0.005 | 0.689±0.004 | - | - | - | 0.726±0.006 | 0.883±0.004 |
| **Best Model** | 0.841±0.003 | 0.715±0.003 | - | - | - | 0.728±0.021 | 0.892±0.001 |
| **Best RF**  **(ifeature, rcpi)** | 0.918±0.016 | 0.818±0.015 | 0.806±0.051 | 0.715±0.106 | 0.750±0.063 | 0.783±0.044 | 0.882±0.026 |

# Table S5. AUPR for ES_pred model on new chemicals and new enzymes

| **Datasets** | **esterase** | **hadsf** | **bkace** | **nitrilase** | **olea** | **fdh2** | **gt** |
| --- | --- | --- | --- | --- | --- | --- | --- |
| **ES_pred** | 0.213 | 0.195 | 0.201 | 0.338 | 0.431 | 0.124 | 0.303 |

ES_pred is a general model for enzyme-substrate interaction prediction developed by Kroll et al [10]. It is trained on a general dataset curated from the Gene Ontology database, UniProt database and Rhea database. Here use the Python scripts to predict the activity relationship (0-1). For chemicals, the SMILES is the input and for proteins the amino acid sequence. The output is the activity probability. AUPR was calculated for the predicted probability versus the true values.

# Figure S1. Top-5 Similarity distributions of proteins and chemicals


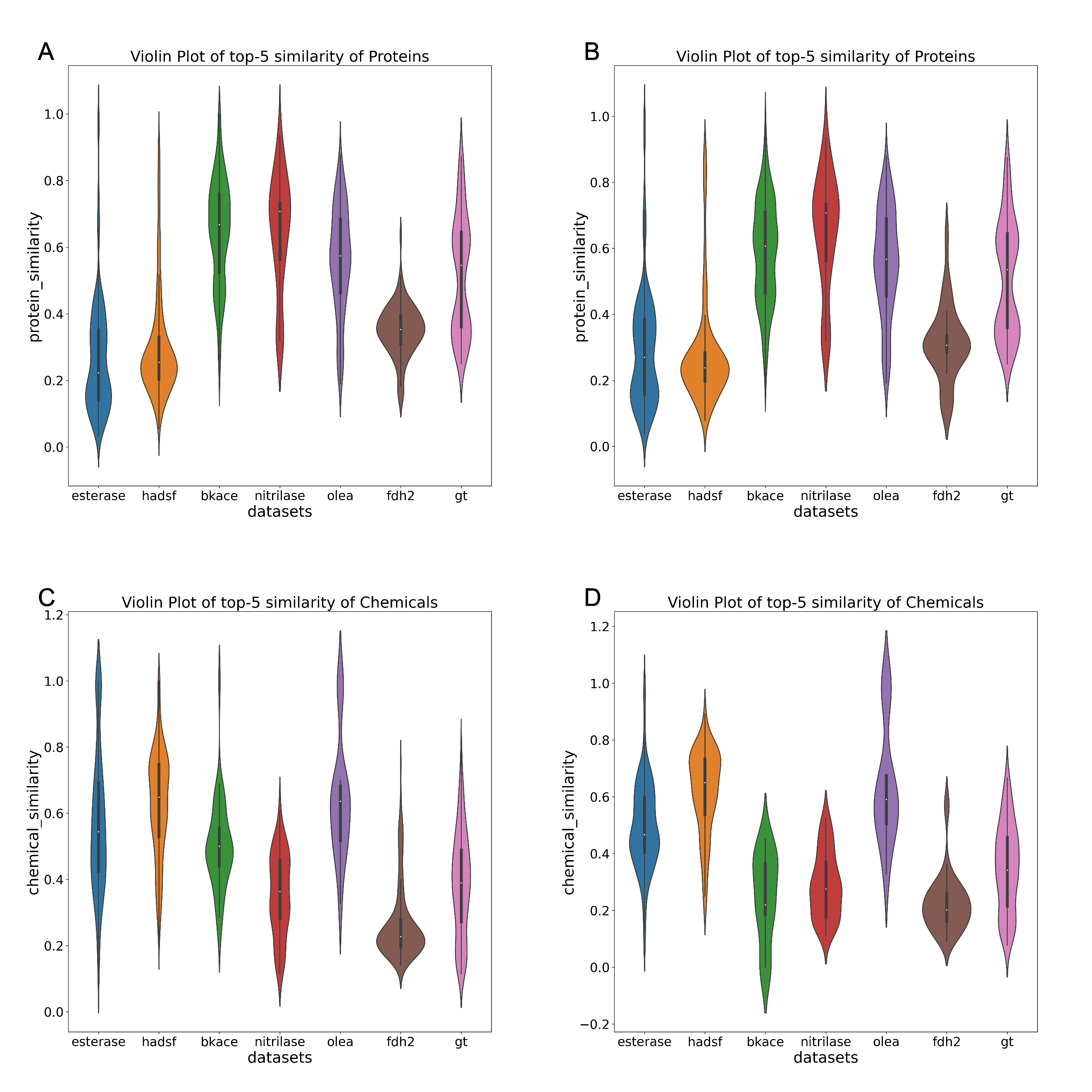


The top-5 sequence similarity and the top-5 chemical similarity were calculated for each dataset as well as for the dataset used for the evaluation of the classification problem. (A) the top-5 protein similarity of the original dataset; (B) the top-5 protein similarity for LCOT; (C) top-5 chemical similarity of the original dataset; (D) top-5 chemical similarity for LPOT

# Figure S2. Metrics for predictions on 7 datasets of LOOT

(A) AUROC for classification; (B) MAE for regression; (C) R2 for regression on positive data; (D) MAE for regression on positive data.


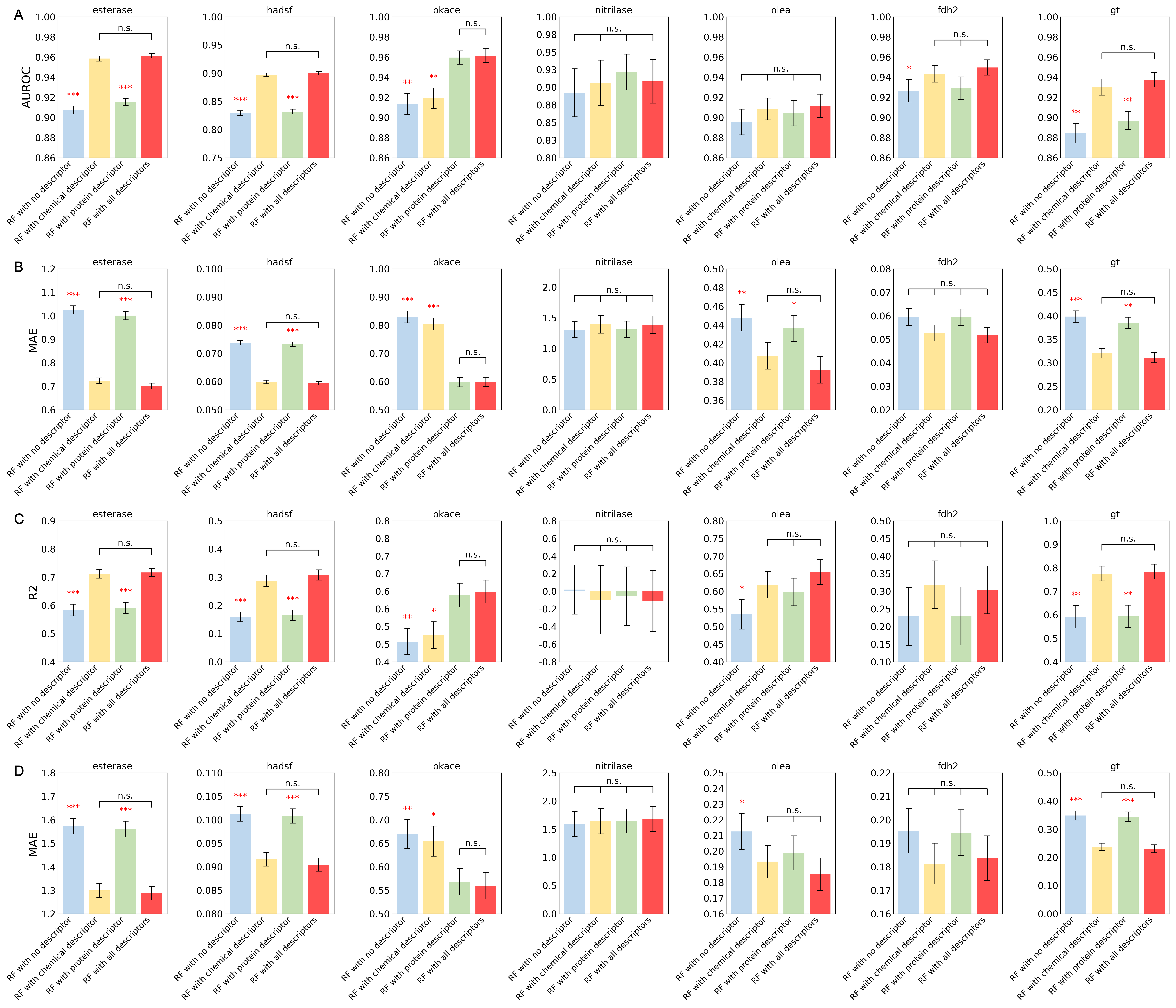


# Figure S3. AUROC for classification on 7 datasets of (A) LPOT and (B) LCOT


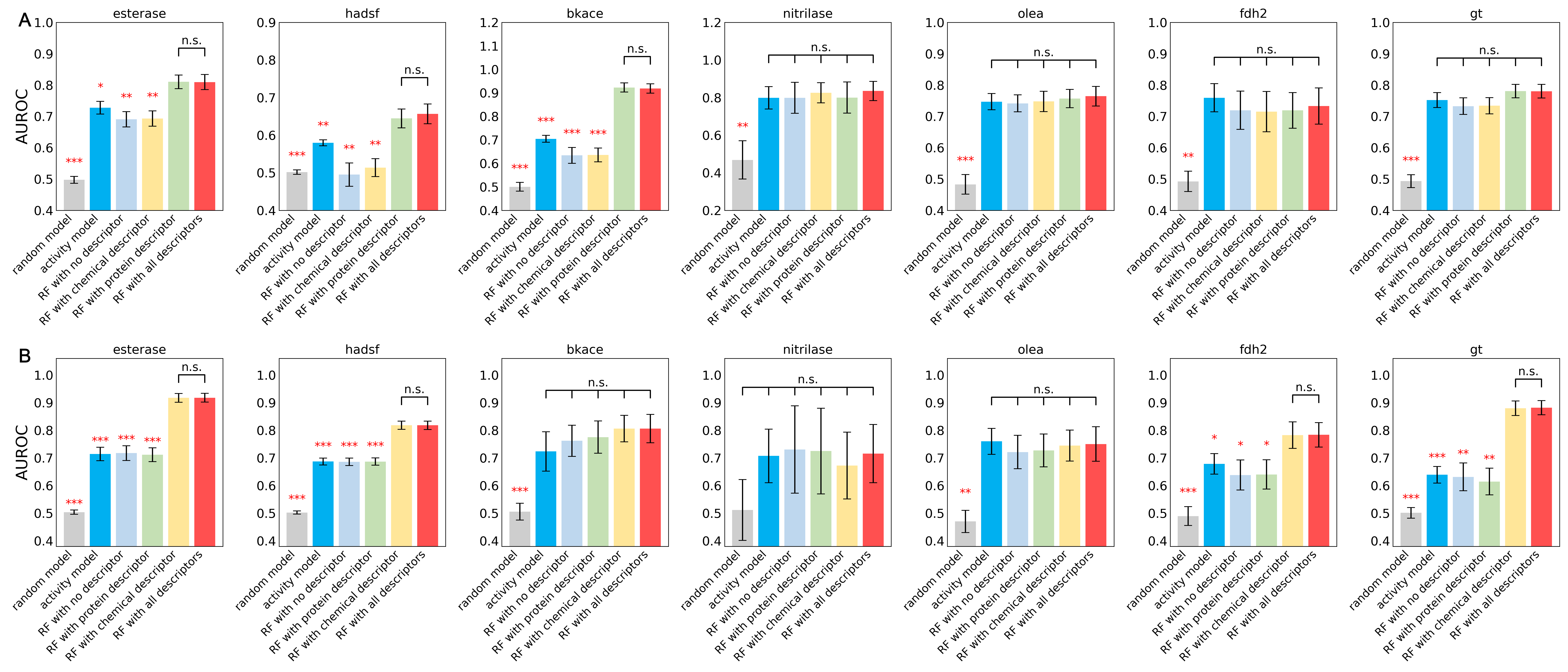


# Figure S4. AUPR for the classification of new enzyme evaluation (LPOT)

(A) nitrilase, (B) olea, (C) fdh2 and (D) gt dataset





# Figure S5 AUPR for the classification of new chemical evaluation (LCOT)

(A) esterase, (B) hadsf, (C) bkace, (D) nitrilase, (E) olea, (F) fdh2 and (G) gt dataset








# Figure S6 AUPR for the classification of new relation evaluation (LOOT)

(A) esterase, (B) hadsf, (C) bkace, (D) nitrilase, (E) olea, (F) fdh2 and (G) gt dataset








# Figure S7. The results of t-SNE for the esterase dataset


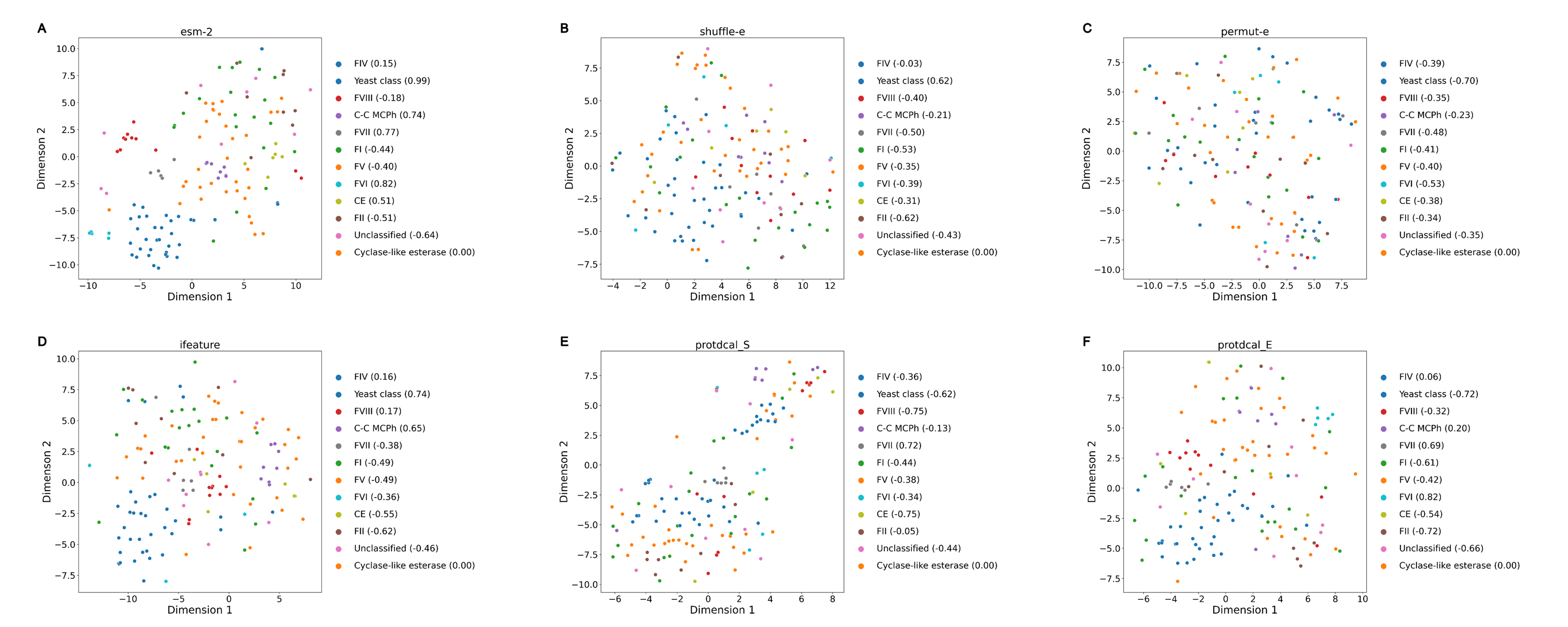


# Figure S8. The results of t-SNE for the hadsf dataset


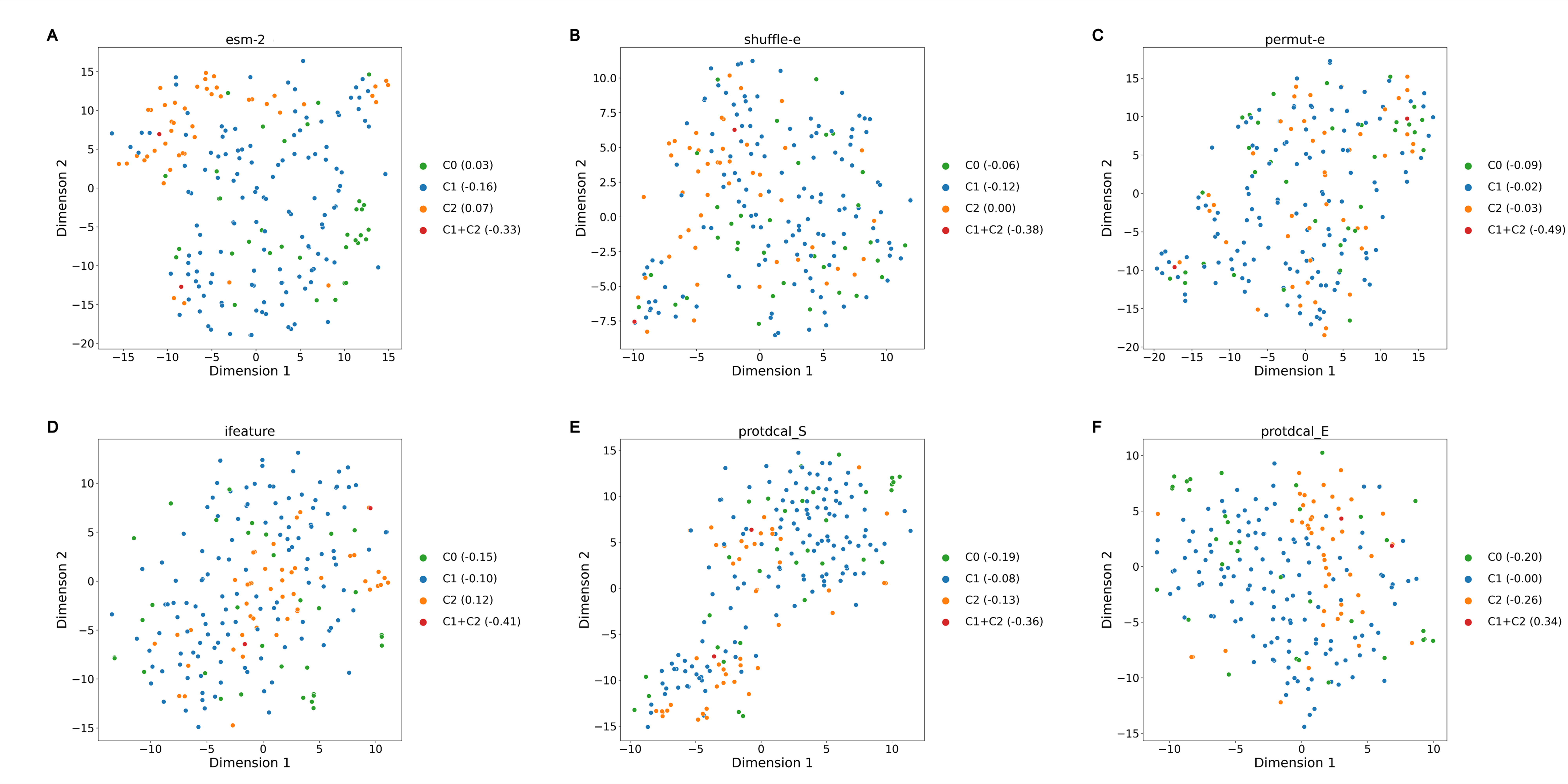


# Figure S9. The results of t-SNE dimension reduction for the nitrilase dataset


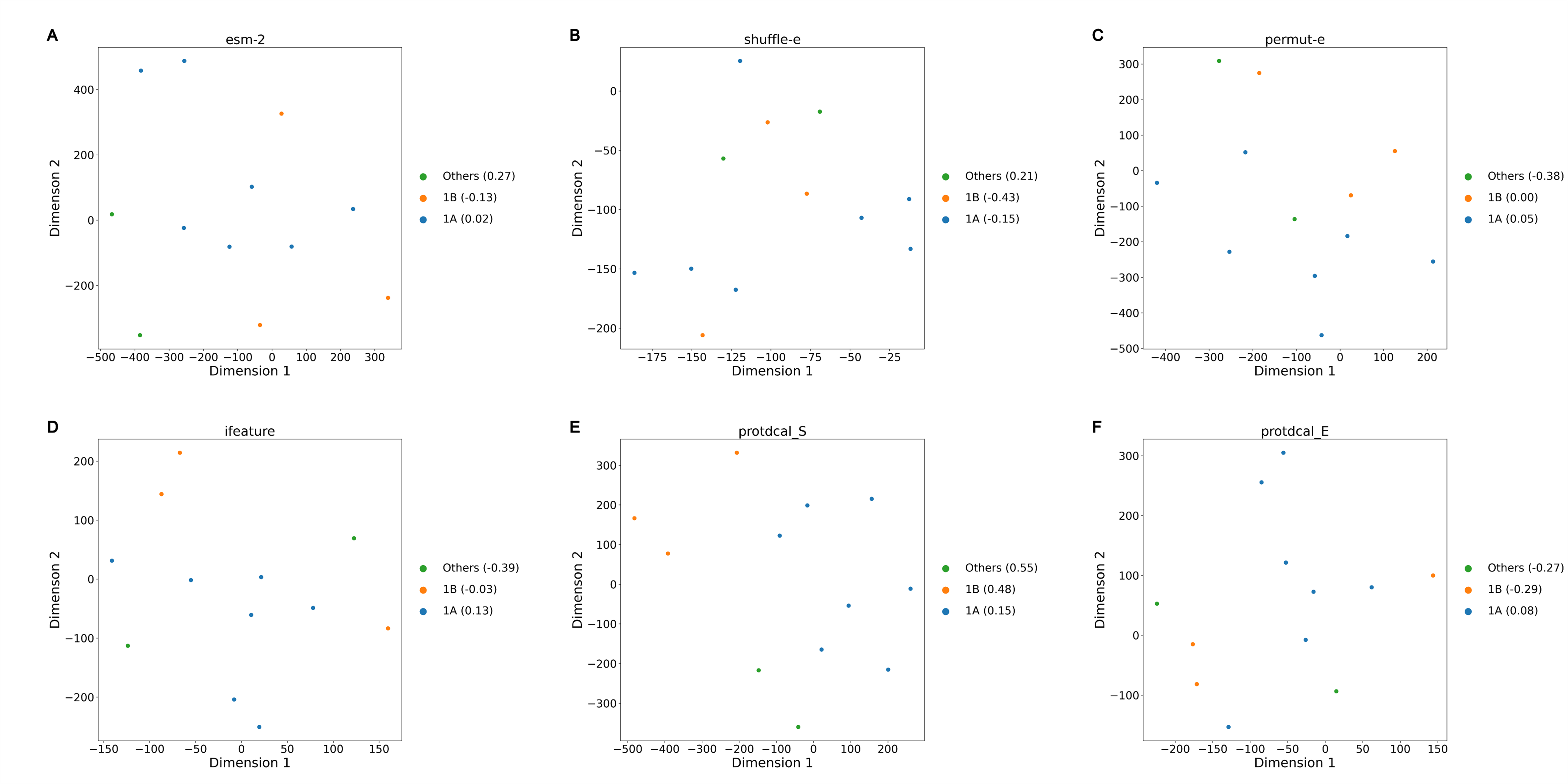


# Figure S10. The results of t-SNE dimension reduction for the olea dataset


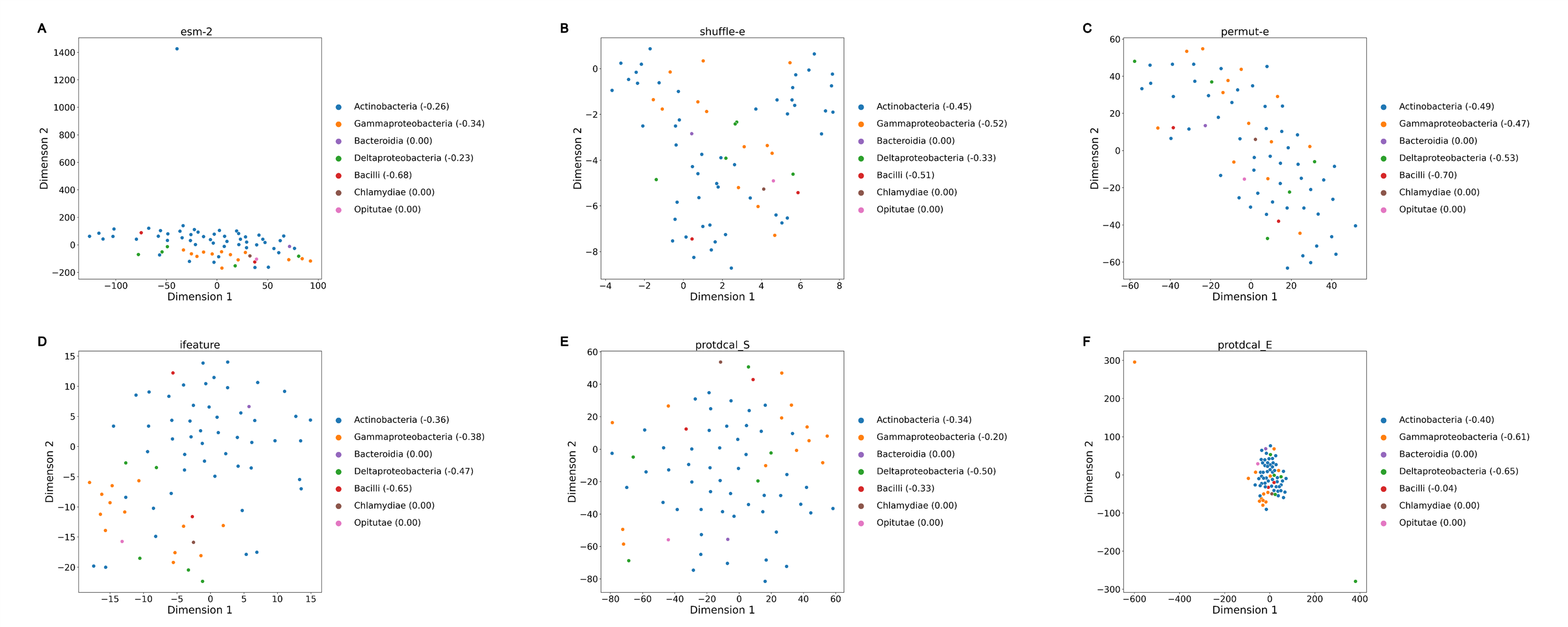


# Figure S11. The results of t-SNE dimension reduction for the fdh2 dataset


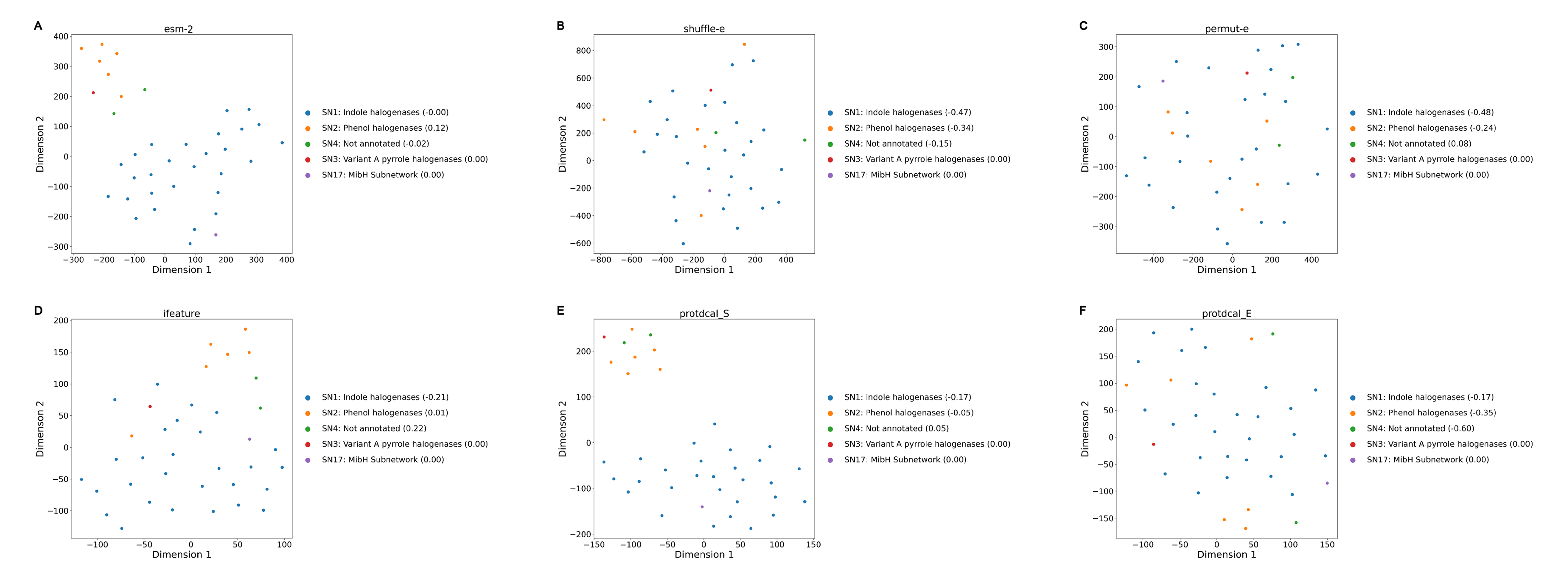


# Figure S12. The results of t-SNE dimension reduction for the gt dataset


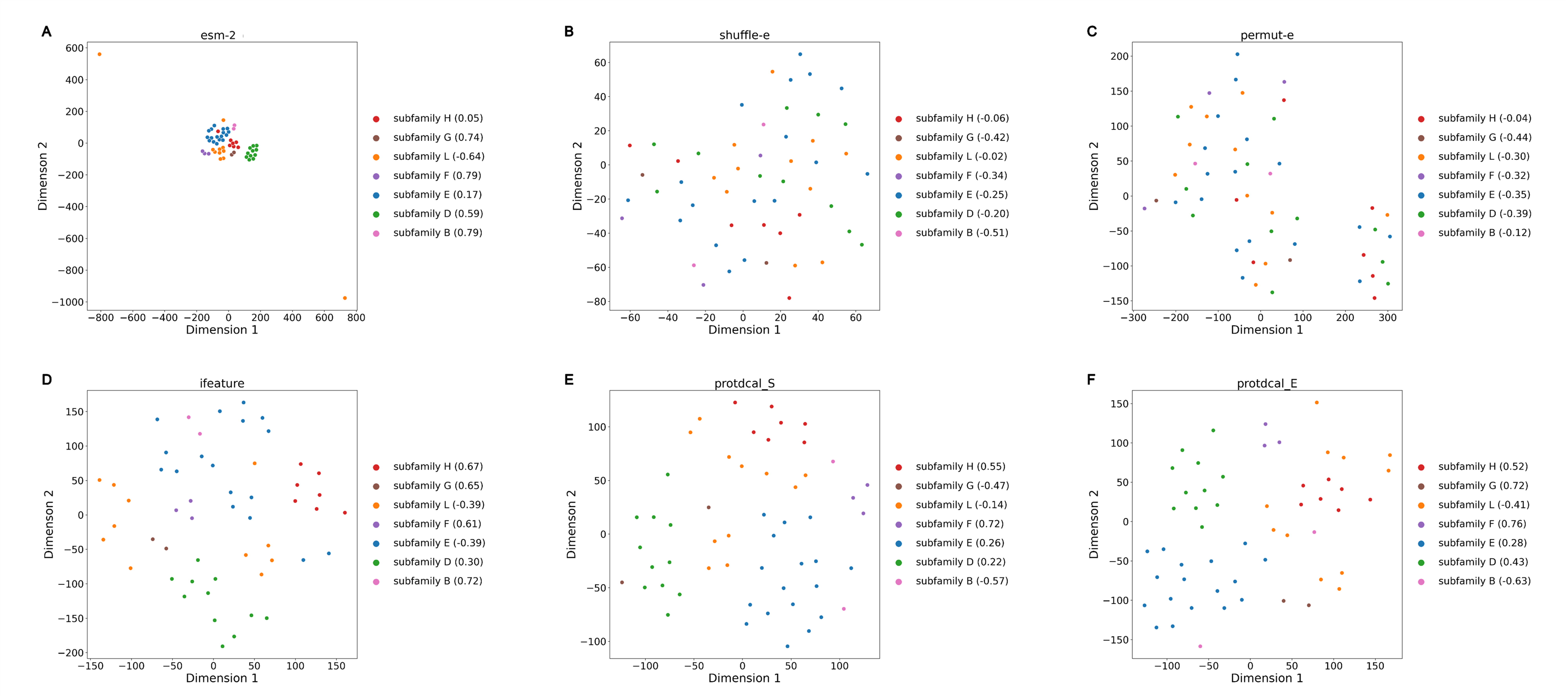


# Figure S13. Correlation coefficients (A) between statistics of datasets; (B) LPOT-AUPR against statistics of datasets; (C) LCOT-AUPR against statistics of datasets


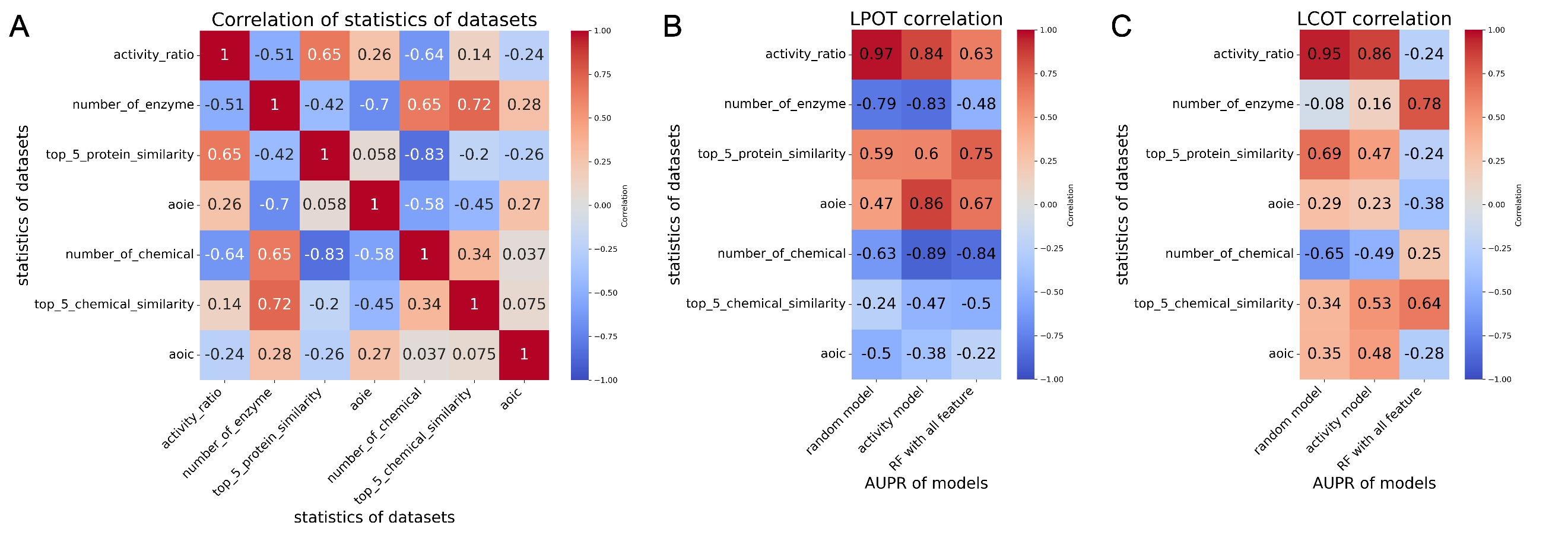


# Figure S14. The correlation of the LPOT-AUPR of RF with all descriptors against statistics of datasets

(A) activity ratio, (B) the top-5 chemical similarity, (C) aoic and (D) the number of chemicals


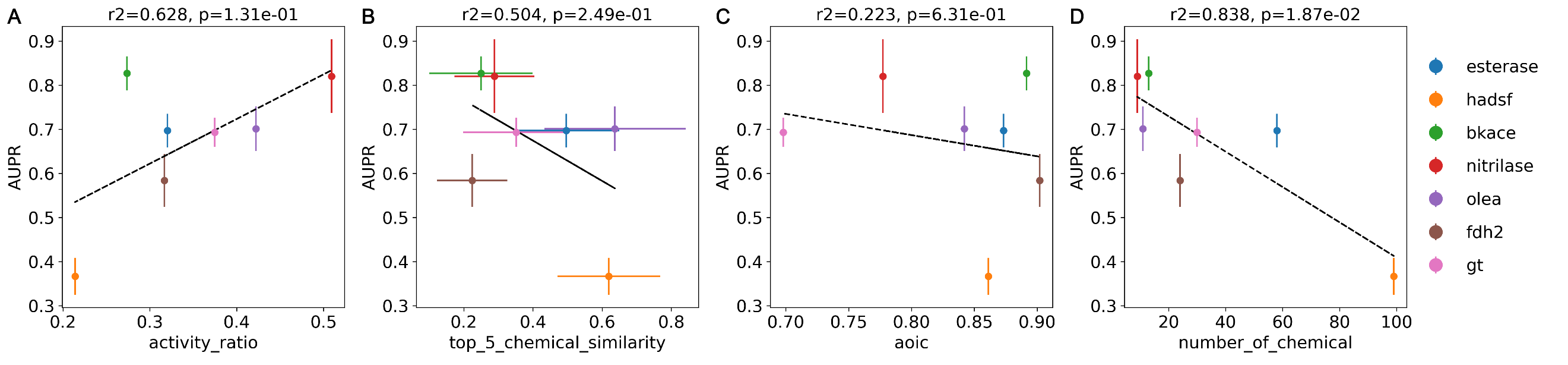


# Figure S15. The correlation of the LCOT-AUPR of RF with all descriptors against statistics of datasets

(A) the top-5 protein similarity, (B) aoie, (C) the number of enzymes, (D) activity ratio, (E) the top-5 chemical similarity, (F) aoic and (G) the number of chemicals


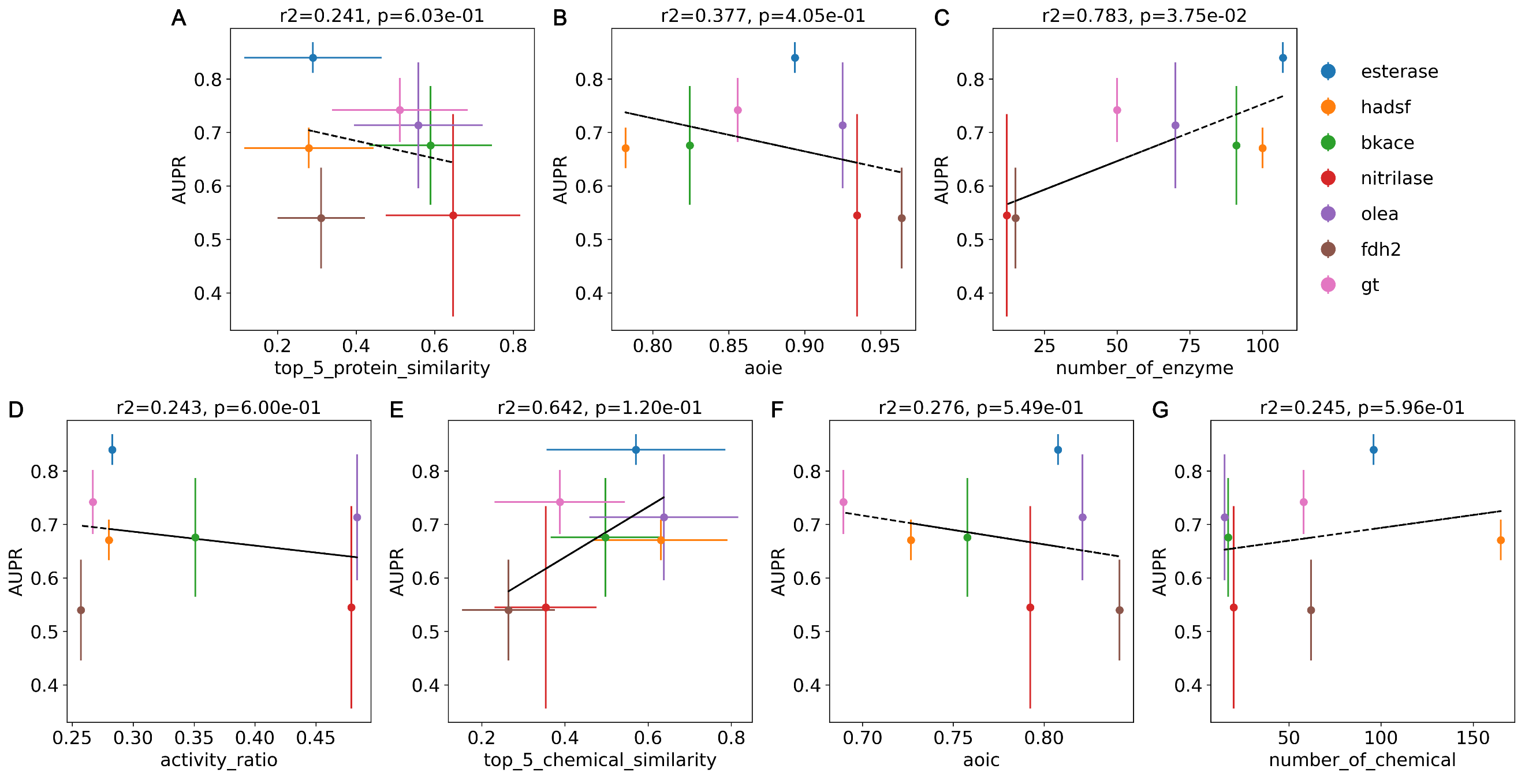


# Figure S16. The distribution of pLDDT scores of ESM-fold *de novo* structure predictions for 7 datasets


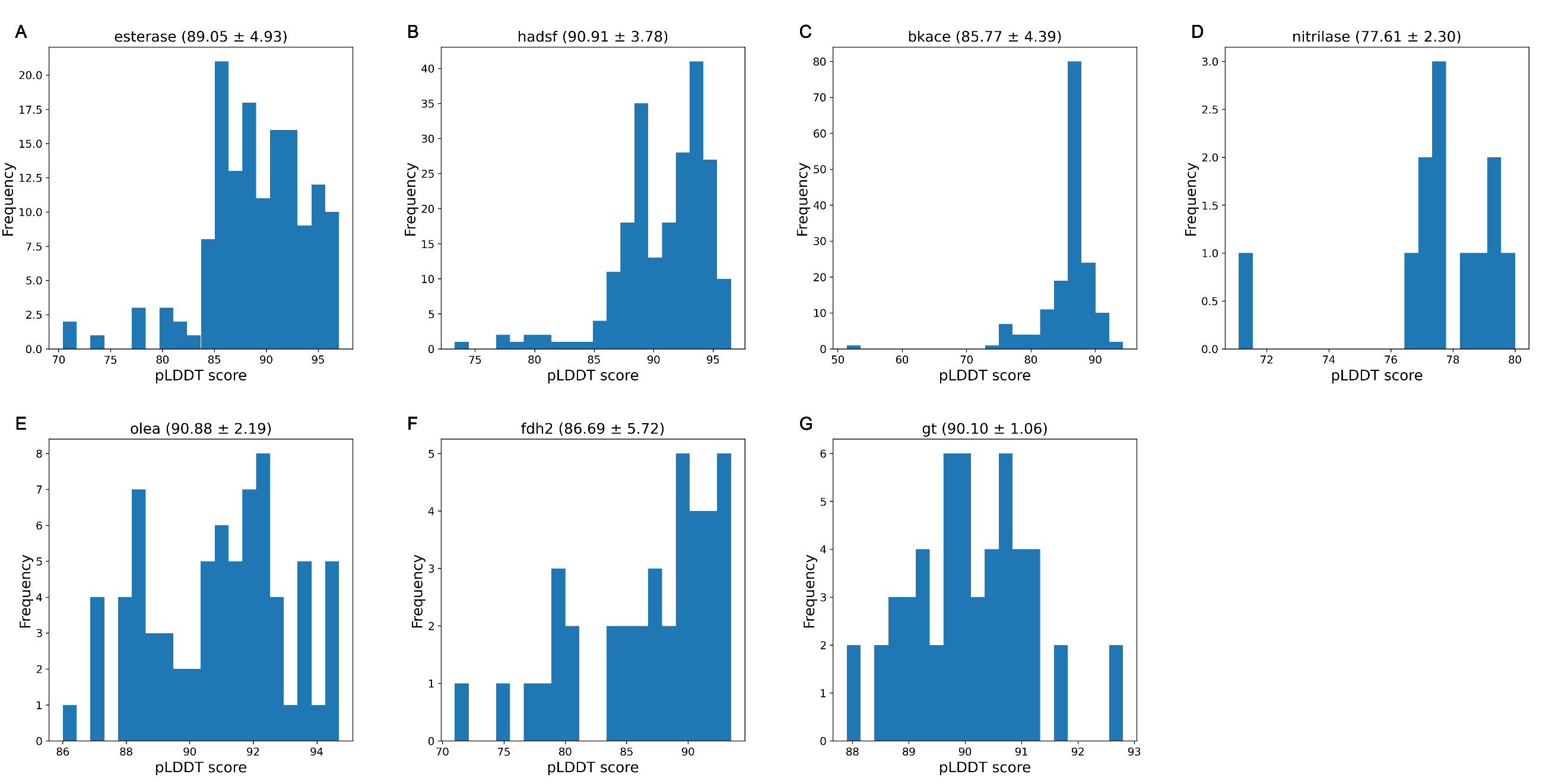


# Figure S17. The distribution of QMEAN Z-scores of Swiss-model templated-based structure predictions for 7 datasets


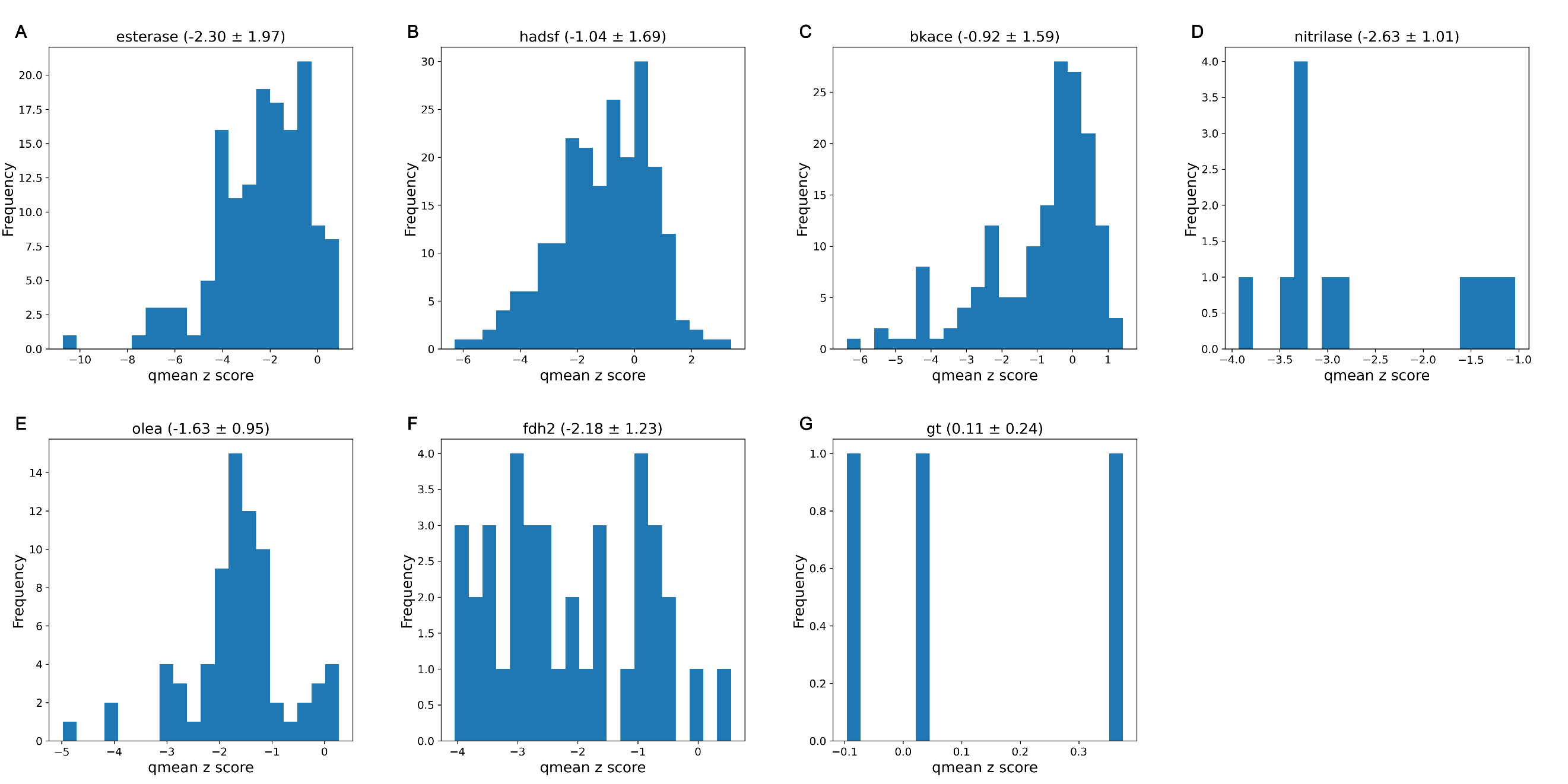


For the gt data set, 50 of the 53 structures came from AlphaFold DB. We believed that these has undergone strict structure inspection. Only three other structures using homology modelling are reflected in the above figure.

# Reference

[1] Martinez-Martinez M, Coscolin C, Santiago G, Chow J, Stogios P J, Bargiela R, et al., Determinants and Prediction of Esterase Substrate Promiscuity Patterns*.* ACS Chem Biol, 2018. **13**(1): 225-34.

[2] Huang H, Pandya C, Liu C, Al-Obaidi N F, Wang M, Zheng L, et al., Panoramic view of a superfamily of phosphatases through substrate profiling*.* Proc Natl Acad Sci U S A, 2015. **112**(16): E1974-83.

[3] Bastard K, Smith A A, Vergne-Vaxelaire C, Perret A, Zaparucha A, De Melo-Minardi R, et al., Revealing the hidden functional diversity of an enzyme family*.* Nat Chem Biol, 2014. **10**(1): 42-9.

[4] Mou Z, Eakes J, Cooper C J, Foster C M, Standaert R F, Podar M, et al., Machine learning‐based prediction of enzyme substrate scope: application to bacterial nitrilases*.* Proteins: Structure, Function, and Bioinformatics, 2021. **89**(3): 336-47.

[5] Kautsar S A, Blin K, Shaw S, Navarro-Munoz J C, Terlouw B R, van der Hooft J J J, et al., MIBiG 2.0: a repository for biosynthetic gene clusters of known function*.* Nucleic Acids Res, 2020. **48**(D1): D454-D8.

[6] Fisher B F, Snodgrass H M, Jones K A, Andorfer M C, and Lewis J C, Site-Selective C-H Halogenation Using Flavin-Dependent Halogenases Identified via Family-Wide Activity Profiling*.* ACS Cent Sci, 2019. **5**(11): 1844-56.

[7] Yang M, Fehl C, Lees K V, Lim E K, Offen W A, Davies G J, et al., Functional and informatics analysis enables glycosyltransferase activity prediction*.* Nat Chem Biol, 2018. **14**(12): 1109-17.

[8] Li F, Yuan L, Lu H, Li G, Chen Y, Engqvist M K, et al., Deep learning-based k cat prediction enables improved enzyme-constrained model reconstruction*.* Nature Catalysis, 2022. **5**(8): 662-72.

[9] Goldman S, Das R, Yang K K, and Coley C W, Machine learning modeling of family wide enzyme-substrate specificity screens*.* PLoS Comput Biol, 2022. **18**(2): e1009853.

[10] Kroll A, Ranjan S, Engqvist M K, and Lercher M J, A general model to predict small molecule substrates of enzymes based on machine and deep learning*.* Nature Communications, 2023. **14**(1): 2787.
